# The value of twinned pollinator-pollen metabarcoding: bumblebee pollination service is weakly partitioned within a UK grassland community

**DOI:** 10.1038/s41598-023-44822-z

**Published:** 2023-10-21

**Authors:** Sandra Ronca, Caroline S. Ford, Joël Allanguillaume, Claudia Szabo, Richard Kipling, Mike J. Wilkinson

**Affiliations:** 1 Department of Life Sciences, Aberystwyth University, Aberystwyth, SY23 3DA UK; 2 Wales Veterinary Science Centre, Y Buarth, Aberystwyth, SY23 1ND Ceredigion UK; 3 Department of Biological, Biomedical and Analytical Sciences, University of the West of England, Coldharbour Lane, Bristol, BS16 1QY UK; 4 School of Computer Science, The University of Adelaide, Adelaide, SA 5005 Australia; 5 The Sustainable Food Trust, 38 Richmond Street, Totterdown, Bristol, BS3 4TQ UK

**Keywords:** Molecular ecology, Ecological networks

## Abstract

Predicting ecological impact of declining bumblebee (*Bombus*) populations requires better understanding of interactions between pollinator partitioning of floral resources and plant partitioning of pollinator resources. Here, we combine Cytochrome Oxidase 1 (CO1) barcoding for bumblebee identification and *rbcL* metabarcoding of pollen carried by bees in three species-rich UK pastures. CO1 barcoding assigned 272 bees to eight species, with 33 individuals belonging to the cryptic *Bombus lucorum* complex (16 *B. lucorum* and 17 *B. cryptarum*). Seasonal bias in capture rates varied by species, with *B. pratorum* found exclusively in June/July and *B. pascuorum* more abundant in August. Pollen metabarcoding coupled with PERMANOVA and NMDS analyses revealed all bees carried several local pollen species and evidence of pollen resource partitioning between some species pairings, with *Bombus pratorum* carrying the most divergent pollen load. There was no evidence of resource partitioning between the two cryptic species present, but significantly divergent capture rates concorded with previous suggestions of separation on the basis of foraging behaviour being shaped by local/temporal differences in climatic conditions. Considering the bee carriage profile of pollen species revealed no significant difference between the nine most widely carried plant species. However, there was a sharp, tipping point change in community pollen carriage across all three sites that occurred during the transition between late July and early August. This transition resulted in a strong divergence in community pollen carriage between the two seasonal periods in both years. We conclude that the combined use of pollen and bee barcoding offers several benefits for further study of plant-pollinator interactions at the landscape scale.

## Introduction

Measures taken to increase in global food production have led to the erosion of biodiversity^1^. The fate of bumblebees has attracted particular attention^2–4^ perhaps in part because their decline has also been linked with reduced food production^5,6^.

The coexistence of sympatric bumblebee species within plant communities partly relies on the extent to which they compete for available food resources. Competition theory dictates that pollinator species coexisting within the same ecological niche must partition resources^7^ or else the less well-adapted species will become locally extinct^8^. At the same time, the plant species they serve are at least partly dependent upon pollinators to ensure adequate seed set and to avoid inbreeding^9^. Thus, the needs of both plants and their pollinators are intertwined^10^.

At a practical level, the ability to study interactions from both perspectives (pollinator to plant and plant to pollinator) requires both a reliable means for accurate bee identification and an effective means of characterising the pollen being moved. This is most commonly achieved through observation of bee foraging in the field, either directly^11–14^ or by video recording^15^. However, *Bombus* species are also noted for convergent colour patterns and high levels of intraspecific variation^12–14^, and so reliable identification of many bee species can be challenging, particularly for cryptic species^14^. The type subgenus *Bombus s.str.* is widespread in Europe and comprises of four morphologically similar species in the UK: *Bombus cryptarum*; *B. lucorum*; *B. magnus* and *B. terrestris*. Of these, only *B. terrestris* can be identified in the field with reasonable confidence, despite a minority of *B. terrestris* workers having been described as phenotypically indistinguishable from other taxa in the subgenus^16^. The remaining three species are notoriously difficult to differentiate and are commonly grouped under one of the following informal epithets: *B. lucorum* species complex; *B. lucorum* s.l. or *B. terrestris* s.l.^17–20^. The group nevertheless represents some of the commonest and widespread members of the genus, and all are important pollinators^21^. Scriven and colleagues^17^ addressed this problem using Cytochrome Oxidase I (CO1) DNA amplicons from tarsal samples to identify bees captured around the UK to a high level of certainty. Information on foraging behaviour was inferred indirectly from where the bee was captured^17,22^.

Characterisation of pollen carriage presents a different kind of challenge. Anatomical diagnosis of pollen recovered from captured bees appears to be the most widely used means of describing pollen carriage by pollinators foraging within a plant community^23,24^. Although the approach is widely used, it is notoriously labour-intensive, prone to operator bias and provides highly variable taxonomic separation of plant groups^25^. These limitations have led to increasing interest in metabarcoding of pollen loads to profile pollen carried by pollinators^26–28^. The premise of this approach is that the number of plant-taxon-specific barcode sequences recovered on a Next Generation Sequencing platform is broadly representative of the number of pollen grains carried by the bee^25,29^ and therefore greatly enhances the resolution of diet reconstruction when compared to the use of anatomical phenotype to separate pollen types^23^.

The current study seeks to explore the value of combining CO1 barcoding for pollinator species identification with pollen metabarcoding to define the interactions between bumblebees and the flowering plants they pollinate in species-rich grassland communities. Specifically, we aim to survey for resource partitioning between co-foraging bumblebee species, seasonal shifts in pollinator service and site-specific effects on pollen carriage. For this, we used CO1 barcodes to identify bumblebee specimens and *rbcL* 454 pyrosequencing metabarcoding to characterise the pollen they carried during the 2008/9 summer seasons.

## Results

### CO1 barcoding of bumblebees

A neighbour-joining tree provided clear separation of CO1 barcode sequences from all 24 species of *Bombus* recorded in the UK, with samples clustering into species-specific clades (Fig. S1). There was 100% bootstrap support for each species-specific clade and each clade contained only the representative barcodes from one species. Thus, the data generated suggested that CO1 reference barcode sequence is able to consistently differentiate between UK bumblebees at species rank (Fig. S1).

**Figure 1:**
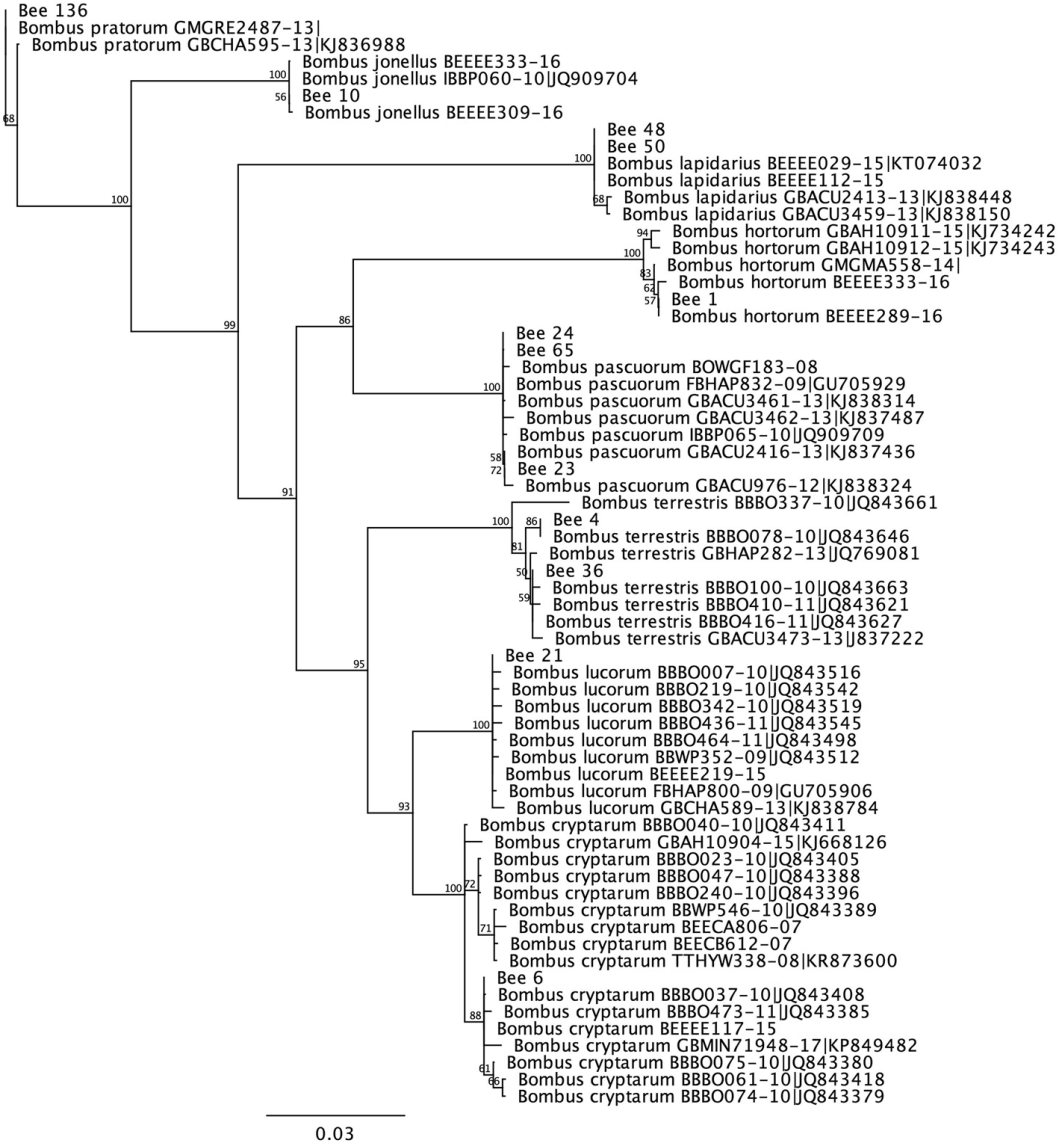
Neighbour Joining tree showing variation in Cytochrome Oxidase 1 (CO1) reference barcode sequences for eight *Bombus* species retrieved from the BOLD Systems V4 database and query CO1 sequences from bees captured at the study sites. Reference barcodes are shown with their BOLD Systems 4 identification code along with query bee barcodes (indicated by the diagnostic bee code number). For simplicity, query bee barcodes with identical sequences are not shown.

We then used the reference barcoding sequence resource to determine the species identity of captured bees. The vast majority (272/288) of bees collected yielded high-quality bi-directional *CO1* DNA barcode sequences. When these sequences were subject to BLASTn searches against the reference barcodes of all UK bumblebee species, the top hits were invariably strong and included several representatives of the same species. When the same sequences were used to assemble a neighbour-joining tree, all query samples from bees captured in the study sites unequivocally aligned to one of eight species-specific clades, again with 100% bootstrap support (Fig. 1). Thus, the CO1 barcode data suggested that we captured eight species of bumblebee, with no ambiguous identifications for any of the captured individuals. These identifications were congruent with the conservative morphological identifications made in the field and subsequently in the laboratory. All specimens morphologically identified as belonging to the *B. lucorum* groups were binned as either *B. cryptarum* (17 samples) or *B. lucorum* (16 samples), but did not include any examples of *B. magnus*.

The unambiguous barcode identification of all specimens to one of eight *Bombus* species allowed us to monitor seasonal changes in bee abundance. When samples were divided into regular collection periods from mid-June through to late August, there was some evidence of divergence between the seasonal appearance of some bee species (Table 1). *B. terrestris* were relatively evenly represented between the early part of the sampling period, June/July (39) and those captured in August (40) (Table 1). By comparison, captures of the two Lucorum species (*B. cryptarum* and *B. lucorum*) were significantly biased towards the early season, with 25 captures between mid-June and July, and only eight in August (Table 1; Chi squared = 6.62, *P* = 0.01). *B. pratorum* showed a more extreme bias towards early capture dates, with all 21 individuals being captured in June/July. In contrast, the vast majority of *B. pascuorum* specimens were captured in August (94/107) (Table 1); again a significant deviation from the even mix seen in *B. terrestris* (Chi squared = 31.3, *P* < 0.00001).

**Table 1:** Summary of bumblebee species captured in three UK Rhos pasture sites over the summers of 2008–2009.

Bee species	Number of bees captured		Total
	June/July	August	
*B. lucorum*	12	5	17
*B. cryptarum*	13	3	16
*B. terrestris*	39	40	79
*B. hortorum*	7	11	18
*B. jonellus*	0	2	2
*B. lapidarius*	1	11	12
*B. pascuorum*	13	94	107
*B. pratorum*	21	0	21

### Flowering resources

In 2009, the total number of flowering heads recorded across the three study sites peaked in mid-July (6544 heads) and then declined to the lowest abundance in late August (563 heads). Across both years, 47 species were sufficiently abundant to be recorded in the transects (Table S1). Combined data revealed roughly equal numbers flowering species from June until the start of August, although the identity of species recorded changed during this time (Table S1). However, there was a notable loss of flowering species during August (Table S1). Overall, there was no significant correlation between the number of bees captured during different parts of the season and either the number of species of flowering plant (linear regression, r^2^ = 0.082, permutation *p* = 0.64, NS) or the total number of flowering heads in the study sites (linear regression, r^2^ = 0.22, permutation *p* = 0.42, NS).

#### RbcL barcoding

The local reference set of *rbcL* barcode sequences were able to diagnose all genera and all species except for the following five sister species pairs: *Stellaria uliginosa/graminae*; *Epilobium montanum/palustre*; *Dactylorhiza maculate/praetermissa*; *Galium palustre/saxatile* and *Ulex europaeus /gallii*.

When the set of reference *rbcL* sequences was expanded to include all species recorded in the surrounding 10 km^2^ landscape, we were able to diagnose around 70% of pollen types (Table 2). This figure included many wind-pollinated species, including the cryptic and semi-cryptic belonging to the *Poaceae* (grasses) and Cyperaceae/Juncaceae (sedges/rushes). However, none of the pollen barcodes recovered from any of these species/species groups exceeded 1% of the total counts recovered, and 270 of the 272 bees carried < 1% of these pollen types, with no bee carrying more than 7% of any wind-pollinated species/species group. These were therefore removed from subsequent analyses, allowing the remaining pollen barcodes to separate between 74 and 79% of the species in the surrounding 10 km square, and 100% of the genera (Table 2).

**Table 2:** Efficacy of *rbcL* pollen species diagnosis.

Site	No of reference barcodes for that 10 km^2^	Percent species diagnosis (%)	Diagnosis of insect-pollinated species (%)
Rhos Glyn yr Helyg (RGYH)	426 (386)	70	74
Rhos Glandenys (RG)	449 (410)	70	74
Rhos Fullbrook (RF)	387 (355)	73	79

Percentage species identification by *rbcL* sequence for all flowering plant species reported within the 10 km^2^ that surrounded each study site. Species discrimination calculated using K2P distances between species.

### Pollen carriage on captured bees

There were 191, 896 sequence reads that passed filter from the pollen samples. When these processed reads were linked to the bee from which they originated using the 454 tags, a mean of 706 pollen barcode sequences were assigned to each of the 272 bumblebees captured. When these were compared with reference barcodes, we presumed that most pollen on bees originated from local foraging. We therefore performed an initial screen matching the pollen-derived 454 sequences to the reference barcodes of plant species found within the study sites. Matches to these sequences accounted for the majority of sequences interrogated, but external (non-study site) pollen species averaged 9.6% of the recovered sequences overall (Table 3). As expected, the imposition of a 5% threshold for a pollen species to be included in the refined pollen profiles of individual bees greatly increased the proportion of local hits and reduced the mean value of external (non-study site) pollen to just 1.6% (Table 3).

**Table 3:** Table showing the percentage of pollen barcodes that matched to reference sequences of plants within study sites or only present in the surrounding landscape (10km^2^).

*Bombus* species	Without 5% threshold		With 5% threshold	
	% barcodes match species within study sites (local foraging)	% barcodes match species outside study sites	% barcodes match species within study sites (local foraging)	% barcodes match species outside study sites
*B. cryptarum*	89.1	10.9	96.9	3.1
*B. hortorum*	91.9	8.1	98.7	1.3
*B. jonellus*	95	5	96.8	3.2
*B. lapidarius*	85.9	14.1	98.5	1.5
*B. lucorum*	91.7	8.3	99.6	0.4
*B. pascuorum*	91.5	8.5	98.8	1.2
*B. pratorum*	87.5	12.5	98.3	1.7
*B. terrestris*	90.7	9.3	99.4	0.6

Figures are presented with and without a 5% abundance threshold for inclusion of plant barcodes in the profiles of individual bees.

### Global analysis of bumblebee pollen loads

Of all sequences that passed filter, at least one of the captured bees carried > 0.99% pollen from 43 of 47 plant species recorded from study sites and from a further 11 species/OTUs from the surrounding 10 km^2^ landscape (Table S2A). This reduced to 37 local species and 3 from the wider landscape when a 5% threshold was imposed for inclusion of a pollen type in the profile of any one bee (Table S2B).

When the carriage profiles of each bee species were considered in isolation, twelve plant species/species groups accounted for 88–97% of pollen carried (Table S3). Some of these twelve OTUs were above threshold (5%) for every individual bee and invariably averaged at or above 1% of pollen carried by the bee species as a whole for all plant species except *Lychnis flos-cuculi* (Table S3). However, at this level of data resolution, there was little evidence of pollen resource partitioning between bumblebee species, except for a modest divergence in the profile of *B. pascuorum* (Table S3). Note that *B. jonellus* was omitted from these and subsequent analyses on the basis of there being only two representatives of this species.

There was considerably more variation revealed when the profiles of individual bees were considered separately, with extensive variation being seen between members of the same bee species (Table S4). All individual bees carried several pollen species and no single pollen species was entirely diagnostic of the pollen load of any one bee species (Table S4). There were nevertheless differences in the variability of pollen profiles carried by the bee species. For example, mean number of pollen species detected on individuals varied from 4.6 pollen species per bee for *B. pratorum* to 9.4 species per bee for *B. lapidarius*. More poignantly, bees in which one pollen species dominated carriage (> 50% of counts recovered from the individual) was significantly more abundant for *B. pratorum* (15/21 bees) than for *B. lapidarius* (1/12 bees) (Chi squared with Yate’s correction = 9.8, *p* = 0.0018)*,* implying a more restricted carriage for *B. pratorum* when compared to *B. lapidarius* (Table S3). Variance in pollen diversity of the other bee species, including the two cryptic Lucorum species was more modest (Tables S2, S4).

There was no clear relationship between the pollen carried by the bees and plant flowering head abundance (Tables S1, S4) and no obvious visual indication of resource allocation between bee species, except perhaps for a stronger preference shown by *B. pratorum* for *Lychnis flos-cuculi* (Table S4). Moreover, only 17 bees carried *Lychnis flos-cuculi* pollen above the 5% threshold, but 12 of these carried more than 80% of this pollen type. The 21 *B. pratorum* bees captured included ten of these twelve individuals (Table S4). Differences between pollen profiles of the other bee species was more subtle and masked by considerable variation between individuals of the same species (Table S4).

We next applied multivariate analyses to seek evidence of resource partitioning between bees. For this, we initially attempted to minimise potential skewing attributable to exponential amplification during PCR by converting the pollen carriage data into binary format. Here, each pollen type was recorded for each bee on a presence/absence basis (1, 0) provided the proportion of pollen present exceeded threshold (set between 1 and 10%). Fifteen pairwise combinations were consistently identified as significantly divergent for all threshold levels but an additional 1–5 pairings were also significant below 6% (Table S5). However, the overall F values generated by PERMANOVA fell beyond 5%. Stress levels of NMDS plots generated from the data were invariably high (> 0.2) but lowest at the 5% threshold (Table S5). The stress values improved when plots were restricted to the significantly divergent species pairings but remained above 0.21 throughout and were again lowest at the 5% threshold (data not shown).

Ternary conversion of the same data using log_10_ intervals starting at the optimum threshold of 5% found with the binary data (i.e. three categories of: 0 =  < 5% barcodes per bee;1 = 5–50% per bee; > 50% count-2) provided similar but only slightly improved pairwise separation of species by PERMANOVA (Table S6). NMDS plots were again suggestive of divergence but failed to provide stress test results below 0.2 (Table S6).

**Figure 2:**
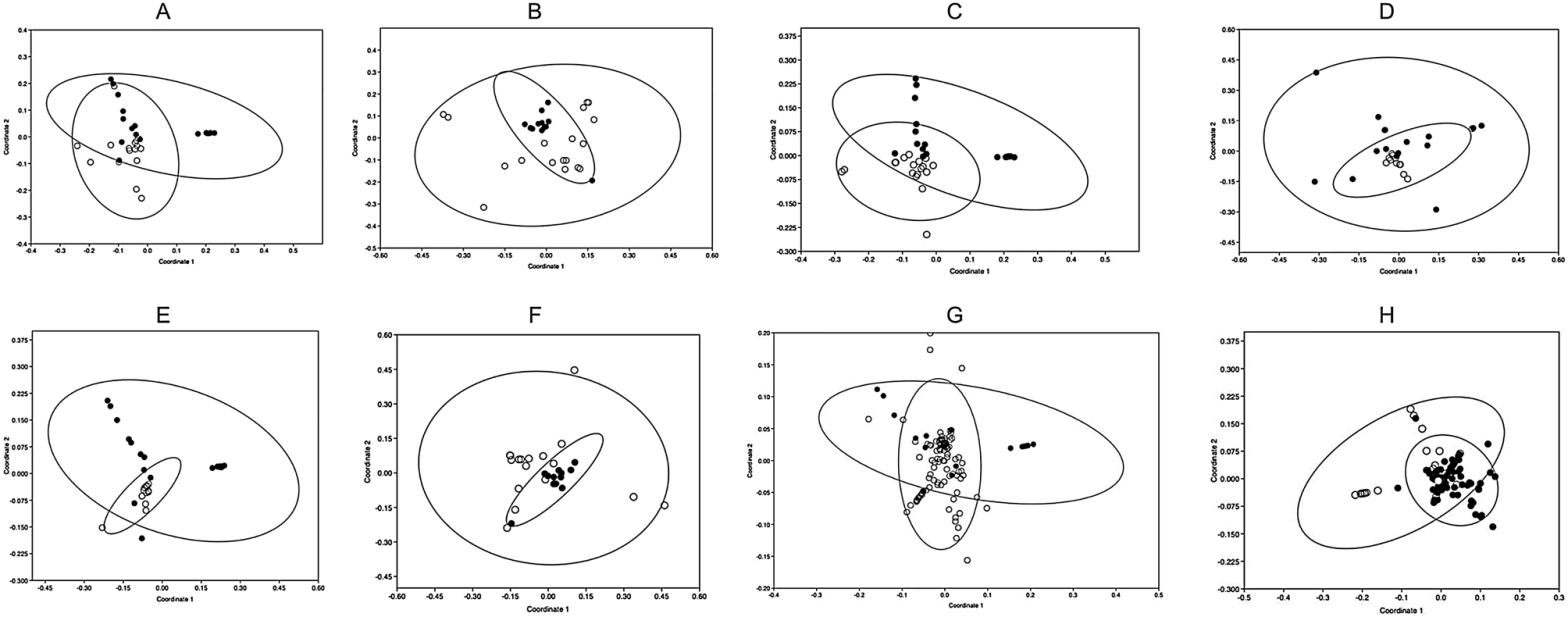
Non-metric Multidimensional Scaling (NMDS) plots of bumblebee pollen carriage. NMDS plots comparing *rbcL* metabarcoding pollen carriage profiles (pollen types above 5% threshold) from *Bombus* species pairs with divergent profiles (Permanova, *p* < 0.05). (**A**). *B. cryptarum* and *B. pratorum.* Stress test = 0.13. (**B**) *B. hortorum* and *B. lapidarius*. Stress test = 0.146. (**C**) *B. hortorum* and *B. pratorum*. Stress test = 0.16. (**D**) *B. lapidarius* and *B. cryptarum*. Stress test = 0.138. (**E**) *B. lapidarius* and *B. pratorum*. Stress test = 0.0948. (**F**) *B. lucorum* and *B. lapidarius*. Stress test = 0.106 . (**G**) *B. pascuorum* and *B. pratorum*. Stress test = 0.199.

Finally, when count frequencies were converted into a quantitative format at the 5% threshold, a similar pattern of pairwise species separation was generated, but featured only ten significant species combinations (Table S7). Eight of the ten pairwise species comparisons generated NMDS plots with acceptable stress values of between 0.095 and 0.17. These plots revealed either modest divergence or a difference in the variability of pollen carriage between the following species combinations: *B. lucorum-B. lapidarius*; *B. hortorum-B. lapidarius*; *B. hortorum-B. pratorum*; *B. lapidarius-B. cryptarum*; *B. lapidarius-B. pratorum*; *B. cryptarum-B. pratorum*; *B. pascuorum-B.pratorum*; *B. pratorum-B.terrestris* (Fig. 2). Thus, six of the eight combinations featured *B. pratorum.* However, there was no evidence of divergence in the pollen carried by the two cryptic species (*B. lucorum* and *B. cryptarum*).

**Figure 3:**
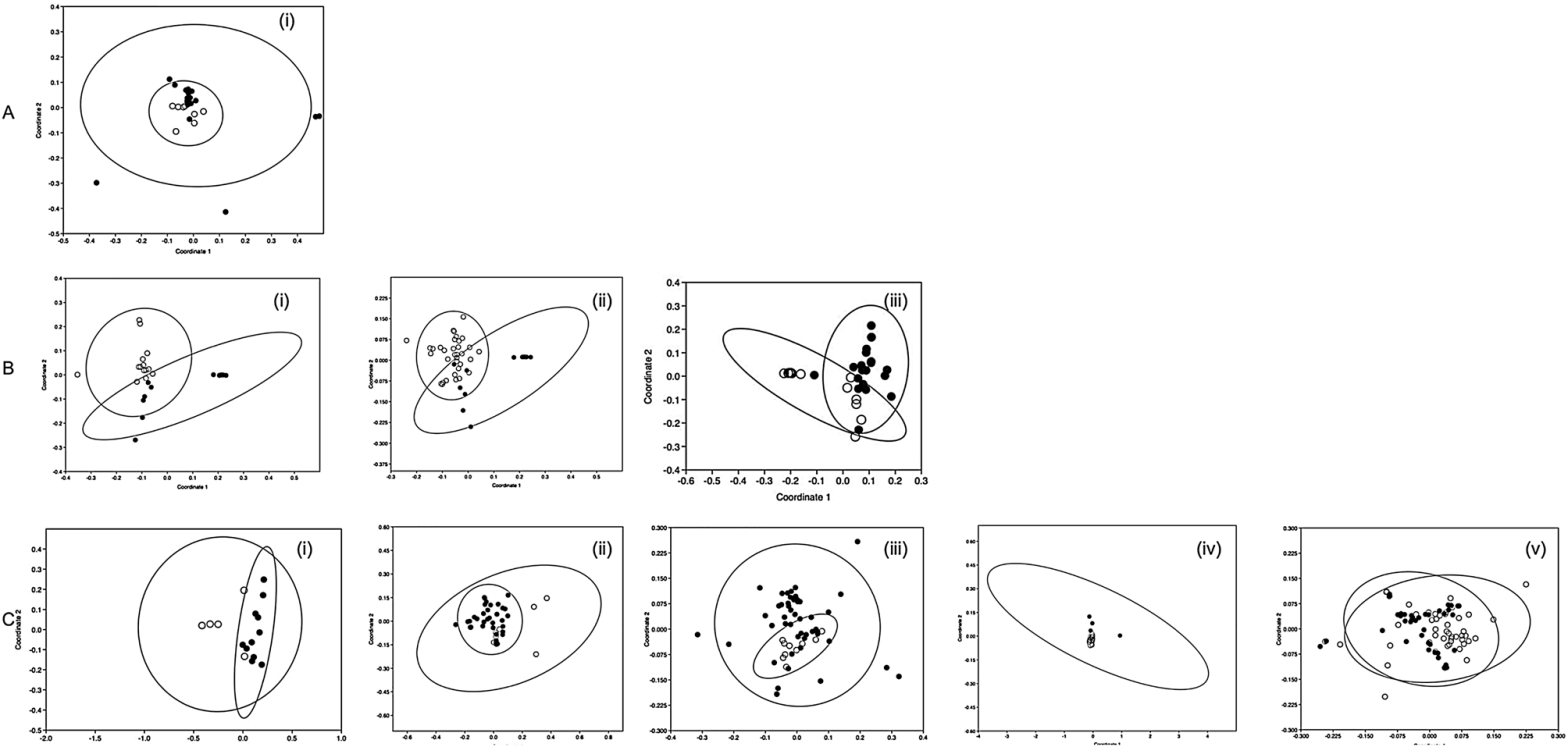
Non-metric Multidimensional Scaling (NMDS) plots of site-specific bumblebee pollen carriage. NMDS plots of species pairings in (**A**) Rhos Fullbrook, RF. *B. pascuorum* and *B. cryptarum*. Stress test = 0.115. (**B**) Rhos Glandenys, RG. i. *B. hortorum* and *B. pratorum*. Stress test = 0.117. ii. *B. pascuorum* and *B. pratorum*. Stress test = 0.189. iii. *B. pascuorum* and *B. terrestris*. Stress test = 0.154. (**C**) Rhos Glyn yr Helyg, RGYH. i. *B. lucorum* and *B. lapidarius*. Stress test = 0.083. ii. *B. lucorum* and *B. pascuorum*. Stress test = 0.143. iii. *B. lapidarius* and *B. pascuorum*. Stress test = 0.137. iv. *B. lapidarius* and *B. pratorum*. Stress test = 0.185. v. *B. pascuorum* and *B. terrestris*. Stress test = 0.153.

We next subdivided the data by location. Quantitative data (5% threshold) collected from the RF site indicated a significant overall effect of bee species on pollen carriage (PERMANOVA, F = 2.035; *p* = 0.0099), but only yielded significant divergence between *B. cryptarum* and *B. pascuorum* (Table S8; Fig. 3A). Counts recorded from the RG site indicated a stronger divergence between species overall (PERMANOVA, F = 4.386; *p* = 0001) and included three divergent species pairings, all involving *B. pratorum*, viz: *B. hortorum-B. pratorum*; *B. pascuorum-B. pratorum; B. pratorum-B.terrestris* (Table S8)*.* NMDS plots of these combinations had stress values in the range 0.11–0.19, with all indicative of at least modest divergence in pollen carriage (Fig. 3B). Evidence of resource partitioning also came from the RGHY site (PERMANOVA, F = 3.292; *p* = 0.0001), with the following five species combinations diverging significantly: *B. lucorum-B. lapidarius; B. lucorum- B. pascuorum; B. lapidarius- B. pascuorum*; *B. lapidarius- B. pratorum* and *B. pascuorum*- *B.terrestris* (Table S8). NMDS plots of these combinations all possessed stress levels below 20% and indicated at least modest divergence in carriage profiles for some combinations (Fig. 3C). The two cryptic species of the Lucorum complex (*B. lucorum* and *B. cryptarum*) failed to diverge significantly in all three sites.

We next sought more subtle evidence of niche separation between *B. lucorum* an *B. cryptarum* based on the seasonal timing of foraging. The date and location of capture of these two species revealed that co-appearance of both species on a collection date-site combination was marginally but significantly lower than expected by chance (Table S9; Fisher’s exact test, *p* = 0.037). This may imply conditions favouring the appearance of each species may differ. We investigated this possibility further by comparing ambient temperatures of the two species when they were captured. This revealed a modest but significant increase in capture rates of *B. lucorum* when temperatures fell in the range 17–19 °C and of *B. cryptarum* in low temperatures (12–15 °C) (Chi squared = 4.20, *p* = 0.041).

### Exploitation of pollinator resources among plant species

**Figure 4:**
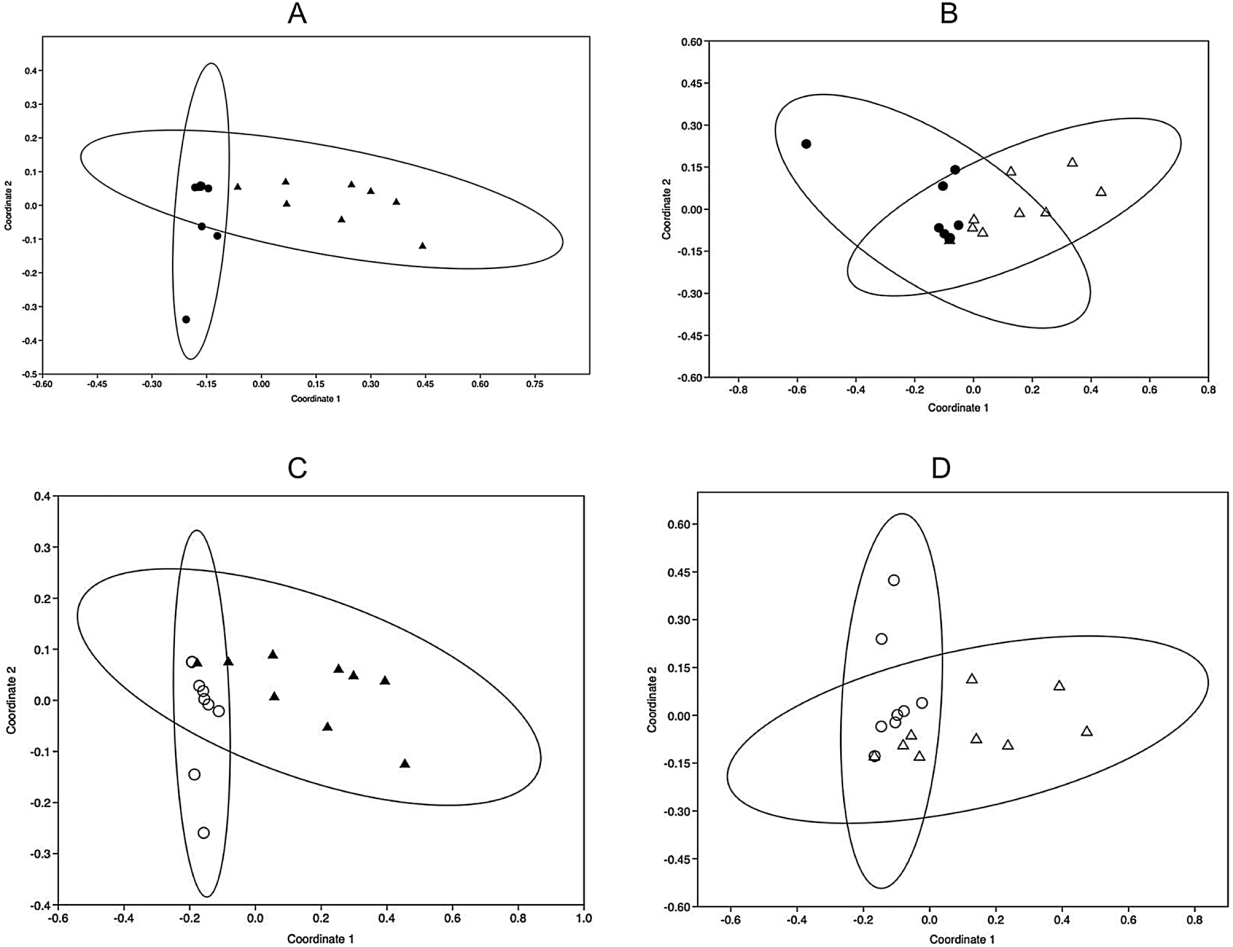
Non-metric Multidimensional Scaling (NMDS) plots of seasonal changes to bumblebee pollen carriage. NMDS plots comparing *rbcL* metabarcoding pollen carriage profiles (pollen types above 5% threshold) on all *Bombus* species captured in (**A**) June (dots) and early August (filled triangles). Stress test = 0.19. (**B**) June (dots) and late August (open triangles) Stress test = 0.2. (**C**) July (open circles) and early August (filled triangles). Stress test = 0.17 (**D**) July (open circles) and late August (open triangles). Stress test = 0.17.

We next sought to compare how pollen from different plant species were carried by the array of bumblebee pollinators present. Summary histogram plots of the nine most dominant plant species (defined as those carrying more than 4.2% of total pollen recorded) revealed some variance in the proportion of bee species carrying different pollen species (Fig. S2) but failed to indicate significant divergence between plant species or collection sites (Table S10). However, when the same profiles were divided by collection date, there was a highly significant seasonal effect and significant diversion between NMDS profiles generated in June and July when compared with those from early and late August (Table S11, Fig. 4A–D).

## Discussion

CO1 DNA barcoding has potential to improve the species identification of bees for field-based studies of foraging behaviour^30^, resource partitioning^31^and ecology^32^, but first requires that species assignment is not compromised by excessive intraspecific variation within the study location. Phylogenetic trees presented in the present study included sufficient reference barcodes of the 24 UK bumblebee species to provide context for both intra- and inter-specific variation of the CO1 locus in the study area. The presence of 100% bootstrap support for all species-specific clades in the tree that combined reference and query barcodes was suggestive that the bees captured did not feature any individuals with ambiguous identity. This premise was supported by strong congruence with the field and laboratory morphological diagnoses, and is concordant with similar studies based on matching high quality, good-length Sanger sequences to a filtered set of full-length reference barcodes held on the BOLD V4 database^33^. These features enabled unambiguous diagnosis of the two cryptic species present (*B. cryptarum* and *B. lucorum*), but concurred with the reported absence of *B. magnum* from mid-Wales^17^.

The barcode-assisted assignment of species identity enabled fluctuations in the visitation frequencies of the different bee species to be monitored in the three sites throughout the study period. Again, the seasonal patterns of bee capture were broadly in accordance with previous reports in the UK, with *B. pratorum* peaking early in the season and *B. pascuorum* being strongly biased towards late season captures in August^34^. Similarly, those species previously identified as peaking mid-season^34^, namely the lucorum group (*B. cryptarum* and *B. lucorum*), *B. lapidarius* and *B. terrestris* all spanned the entire collecting period. The one minor exception was *B. hortorum*, which is recognised as an early season species^34^, but was absent in June and peaked in August.

There are other factors that could also account for the interspecific differences in capture rates found in our study sites. For example, several works have reported differences between bumblebee species in the criteria used to select foraging sites^35,36^. No clear correlation was found in the present study between the abundance of bees and either floral abundance or the number of flowering plant species present, but it is nevertheless plausible that another aspect of fluctuating floral resources such as pollen or nectar yields could be at least partly responsible for the variable bee capture rates observed across the season. Such changes would occur regardless of the broader population dynamics of the species at a regional level. Thus, local capture rates are not a simple reflection of the relative abundance of each species. Other factors could similarly affect site capture rates, including known differences between bee species in nest density and foraging range^37^; in the size of bees^38^; predator threat^39^ or variable learning performance^40^. Differential foraging distances has particularly emerged as a potentially factor in shaping niche differentiation between co-foraging bumblebee species^41,42^. Westphal and colleagues^42^ noted that *B. pratorum* typically foraged over the shortest distances (250 m) whereas *B. terrestris* and *B. lapidarius* travel the furthest (3000 m), which could potentially lead to different patterns of community visitation as found here. Clearly, there are numerous elements that would need to be considered before gaining a predictive understanding of the patterns observed here and elsewhere. In this context it is perhaps interesting to note that we found no indication of any seasonal separation between the cryptic species *B. cryptarum* and *B. lucorum*. This finding concurs with previous works on the species^17,22^ and implies the driver of any ecological separation between these species probably resides elsewhere.

Resource partitioning provides an important driver of niche differentiation between sympatric bumblebee species^43,44^. The application of pollen metabarcoding to characterise pollen carriage has attracted interest^45^, but is predicated on the presumption of at least a semi-quantitative relationship between abundance of a particular pollen species and frequency of barcode counts recovered from that species. There is surprisingly little empirical evidence of a direct relationship between PCR amplicon abundance and number of pollen grains used as a template. In a rare exception, Ronca and colleagues^46^ found significant linear correlation between qPCR Ct values and the quantity of pollen grains in a standard dilution series. Nevertheless, there remains a realistic prospect for confounding variability when comparisons are made between more distantly related taxa. In the present study we initially attempted to address this issue by removing the quantitative element of the data set by compressing the barcode frequency information into a simple binary format (present above threshold or absent). This strategy indicated a significant divergence between pollen profiles carried by different bee species, but lacked sufficient information content to produce NMDS plots with stress test values below 0.29. The nature of interspecific carriage differences was therefore uncharacterised. Consistency in the identity of significantly divergent species pairings when the binary compression of data was relaxed to three categories implied only modest skewing attributable to PCR amplification. Likewise, there was similar concordance when sequence count data was allowed to become quantitative above 5% threshold, albeit featuring fewer species pairings. This move also increased information content sufficiently to allow interrogation using NMDS plots of eight species combinations identified as divergent by PERMANOVA. *B. pratorum* dominated these divergent combinations. This species was also notable in being present only during the early season captures and in possessing the least varied carriage of different pollen types. Divergent bumble bee species pairings that could be characterised by NMDS was restricted to the RGHY site and only included the following combinations: *B. lucorum-B. lapidarius; B. lucorum- B. pascuorum; B. lapidarius- B. pascuorum*; and *B. pascuorum*- *B.terrestris*. Thus, overall evidence of divergence in pollen carriage appeared to be at least partly site-dependent. In agreement with several previous studies on resource partitioning in Bombus^43,47^ although short-tongued species (*B. terrestris; B. lapidarius; B. pratorum; B. lucorum)* and their long-tongued counterparts (*B. pascuorum; B. hortorum*) did feature in the divergent species pairings, they only accounted for around half of those observed in the study. It was therefore not the only factor of importance. We also failed to find evidence of pollen carriage partitioning between the two cryptic species in any site. However, we did find marginal differences in capture rates of these two species, with more *B. lucorum* captured at higher temperatures and *B. cryptarum* dominating when temperatures were cooler. This finding is congruent with an earlier study which revealed that mixed populations of the three cryptic lucorum species (*B. lucorum, B. cryptarum and B. magnus)* are largely restricted to the north and West of the UK^22^, and that “*B. lucorum* is adapted for activity in warmer sunnier conditions, whereas *B. magnus* and *B. cryptarum* are adapted to forage in cooler cloudier conditions”. The same study noted that *B. magnus* has a strong preference for heathland, perhaps providing one possible explanation for the absence of this species from the current study. One intriguing possibility from these findings is that niche separation of these species could be based on foraging according to local ambient temperature conditions, and so could feasibly separate along altitudinal/temporal basis. Testing this possibility would clearly require further work.

Profiles of the bumblebee pollinators that carried the nine most numerous plant species did not provide clear statistical evidence of differentiation between plants. However, these pollinator representation profiles changed sharply during the transition between early (June-July) and late season (August) for all plant species, with a scale of change suggestive of a tipping-point restructuring of the guild composition at this time point across all three sites. The drivers for this transition are not obvious and require further investigation.

Our study found no obvious relationship between global floral abundance and diversity in the source community and the pollen profile carried by the bumblebees. This accords with the relatively few previous works that address this issue^28,48^. Closer examination of the food resources available to the bees in our study revealed a gradual phenological progression in the identity and abundance of flowering plant species throughout June and July, but with a slow decline in flowering abundance and diversity during August. This decline does not fit with a sharp tipping point observed in pollinator use at the start of August but did broadly coincide with the shift in the profiles of bumblebee species captured in these sites. Most bee species were more abundant in the early season, when flowering resources were slightly more abundant and diverse. Moreover, the sharp turnover in foraging bee species occurred at the July–August boundary, and appeared to coincide with the strong divergence in pollinator profiles exhibited by the commonest flowering plant species present. Given the lack of divergence between profiles of plant species, these findings appear to be most consistent with flowering time being the strongest determinant of bumblebee pollen carriage service available for these plant species. Again, this possible explanation requires further investigation.

As with preceding works on Bombus^45,48,49^, we found considerable variation in the diversity of pollen carried by individuals of every bee species. Here, as with previous works^45,48,49^ divergence between the pollen carried by different bee species appears to be subtle and prone to both temporal and spatial variance. There is similarly no clear divide between wind- and insect-pollinated plant species, with the former commonly featuring in the diet of bees, as noted at low frequency here, but reported to be more substantial in studies of early season bee foraging^17,50^. Gaining deeper understanding of the many drivers that shape the patterns of pollen carriage and delivery requires the ability to monitor multiple variables across several spatial and temporal scales. Our contention is that combined use of pollen and pollinator metabarcoding provides an additional tool that may help realise the growing appetite for gaining a systems level understanding of pollinator service provision for plant communities^51^, particularly when viewed in the context of changing land use^52^ and climate change.

At the most basic level, insect-mediated pollination facilitates more efficient transfer of pollen between member of a plant species than is possible through wind. Pollen delivery by pollinators reduces inbreeding and for self-incompatible species, ensures reproductive success. However, this role is to some extent compromised when a pollinator visits multiple plant species in the same community, as this increases the likelihood of interspecific pollen delivery (delivery failure) and reduces the proportion of pollen that is carried by each pollinator (reduced pollen acquisition). In this context, our finding that all individual bees of all *Bombus* species carried multiple pollen species on their bodies implies that all plant species within these communities, even specialists, must experience significant instances of interspecific pollen delivery and competition for pollen acquisition. These observations accord with previous reports based on foraging observations^10^, and also by the characterisation of pollen loads by anatomical examination/metabarcoding^53,54^ It therefore seems plausible that inefficiencies imposed by non-target delivery may be ubiquitous except for extreme specialist associations, but are perhaps outweighed by a net improvement in delivery over passive dispersal systems. However, the relationship between efficiency of intended pollen delivery on a per visit basis, and the overall efficacy of some pollen being delivered to target is not simple^55^. Indeed, a positive relationship exists between overall effectiveness of pollination and the overall frequency of visitations and is predicted to improve as variance in visitation frequency increases relative to conspecific delivery rates^55^. Viewed in this way, floral fidelity may be less important than visitation frequency in determining effective pollen delivery. This then, highlights a key limitation of the present work since fuller understanding would require information of visitation rates (foraging behaviour), pollen carriage and pollen disposition (delivery) rates.

Future efforts to quantify such effects clearly present a significant challenge. However, the relationship between foraging and pollen acquisition is neither direct nor simple, with the most frequent floral visitors not necessarily being most effective pollinators^24^. The combination of pollen metabarcoding, floral resource assessment and *Bombus* barcoding as used in this study represents only part of the way towards addressing this issue. We contend that there is need for further integration of the various aspects of the process, including the study of pollen delivery onto the stigmatic surface, if we ultimately aim to characterise efficacy of pollen movement at the landscape scale.

## Methods

### Survey sites

Three study sites were surveyed in Ceredigion (Wales) from mid-June to the end of August 2009, and in August 2008. All three sites were wet, unimproved, relatively species-rich, grazed pastures, owned and managed by the Royal Society for the Protection of Birds (RSPB) (Table 4).

**Table 4:** Summary of study sites used.

Site	Site type	Grid Ref	Size	Management	Notes
Rhos Fullbrook (RF)	Wet	SN 667,628	2.2 ha (5.9 acres)	Grazing by two ponies, from mid summer	Traditionally managed marsh grassland with some small open heath type areas. Extensive areas of tall reeds
Rhos Glandenys (RG)	Wet	SN 538,650	2.4 ha (5.9 acres)	Grazing by four ponies, from mid summer	Traditionally managed marsh grassland with large stands of reeds but also fairly extensive open grassy areas
Rhos Glyn yr Helyg (RGYH)	Wet	SN 496,514	15 ha (37 acres)	Periodic extensive grazing by cattle through summer	Traditionally managed marsh grassland with clear divide between areas of marsh and thick reeds, and large open grazed areas

#### Phenological progression

The abundance of flowering heads was estimated at 2–4 weekly intervals throughout August 2008, and from mid-June to the end of August 2009 (Table S12). We used the strategy described by Lunt^56^ to characterise changes in floral phenology. Briefly, all open flowers were recorded along a crenellated transect comprising of 10 m sections between two fixed points in at each site (100 m total transect length). Plant species were identified according to Stace^57^.

### Assembly and comparison of CO1 reference barcodes for UK Bombus species

We assembled a set of reference CO1 barcodes for all species of *Bombus* reported to occur in the UK^58^ from the BOLD Systems V4 database (https://v4.boldsystems.org/, last accessed Sept 12^th^ 2022). All barcodes retrieved were aligned and trimmed to a common length (497 bp) using and duplicated sequences removed. The remainder were then used to compile an unrooted neighbour-joining tree using Geneious software v 8.1.8 (www.geneious.com).

### Bumblebee sampling

We followed the method of Memmott^59^ to estimate bumblebee species abundance on each site visitation. Bumblebees were captured along three linear transects per site, totally approximately 100 m (one central transect and two parallel transect 5-10 m from the site margins) for 30 min between 12.00 and 13.00 h on each collection day. Bee sampling coincided with plant survey on dates listed in Table S12. During the survey, individuals matching *Bombus* phenotypes from species known to occur in the location were captured directly from flowers into a plastic container. One individual was captured per container to avoid cross-contamination of pollen loads and particular attention being made to prevent pollen removal from the flower. After collection, samples were snap-cooled on dry ice to immobilise the insect and reduce the chance of pollen becoming dislodged by excessive movement. Samples stored at −80 °C until required.

### Isolation of pollen from stored bees

Individual bees were placed into a 2 ml eppendorf tube containing 1 ml Nuclease-Free water, vortexed and spun at 5000 rpm for 10 min. Supernatant and the bees (and any detached limbs) were removed. The pollen precipitate was then resuspended in Proteinase K solution (275 μl digestion buffer [2% SDS, 100 mM TRIS, 20 mM EDTA, adjusted using 1 M HCl to a final a pH = 8,] and 25 μl (500 u) Proteinase K (Sigma, UK) and used immediately for DNA extraction (see below). No attempt was made to separate corbicular pollen balls from the bees or from detached bee limbs upon removal.

### *CO1* DNA barcoding of bumble bees

DNA was extracted in 2009 from both forelimbs of each bumble bee using the Wizard SV 96 Genomic DNA purification System (Promega, UK) according to manufacturers’ instructions. PCR amplification was performed within a month of extraction using the following universal primers for *CO1*^60^:

LEP(F1), 5´-ATTCAACCAATCATAAAGATATTGG-3´:

LEP(R1), 5´-TAAACTTCTGGATGTCCAAAAAATCA-0.3´.

PCR conditions comprised: 5 cycles of 94 °C for 120 s; 45 °C for 40 s and 72 °C for 60 s, followed by 35 cycles of: 94 °C for 40 s; 51 °C for 40 s and 72 °C for 60 s, with a final 5 min extension step at 72 °C.

Strong amplicons of the appropriate size were subjected to DNA sequencing using the *CO1* barcoding sequencing protocol as described by Hebert et al.^60^. The resultant clean sequences were trimmed to 497 bp (the maximum conserved length across all samples) using Clustal software.

### CO1 barcode identification of captured bees

Trimmed *CO1* sequences data from captured bees were then used for bumblebee species identification. Provisional identification was assigned by BLASTn searches of each query bee sequence against the trimmed reference *Bombus* sequences from the BOLD Systems V4 database (http://www.boldsystems.org/ last accessed 4/2/2022). Identification was verified when query sequences and reference barcode sequences for all 24 UK *Bombus* species were used to compile a neighbour-joining tree on the Geneious software package. Query sequences appearing in monospecific clades containing 100% bootstrap support in the resultant tree were deemed to belong to that species.

### Generation of a reference set of local *rbcL* plant barcodes

We retrieved reference barcodes of all plant species present in the study sites from UK plants listed in the BOLD Systems V4 database (http://www.boldsystems.org/ last accessed 4/8/2021). This set constituted the local reference barcodes.

In addition, we also identified all flowering plant species recorded within the 10km^2^ UK national grid square containing each of the three collection sites using the online atlas of the UK and Irish flora (https://www.brc.ac.uk/plantatlas/ last accessed 4/8/2021). This represented the regional reference barcodes.

### DNA extraction from pollen load samples

Pollen samples were resuspended in aliquots containing 275 μl digestion buffer (2% SDS, 100 mM TRIS + HCl to give a pH = 8, 20 mM EDTA) and 25 μl (500u dissolved in nuclease-free water) Proteinase K (Sigma, UK). Samples were incubated at 65 °C for 15 min under constant agitation. After incubation, DNA was isolated from pollen using the Qiagen DNeasy Plant Kit (Qiagen UK) according to manufacturers’ instructions.

The following universal *rbcL* primers were used to generate amplicons:

F 5´ATGTCACCACAAACAGAGACTAAAGC 3´ (Kress and Erickson^61^).

R 5´AGTCCACCGCGTAGACATTCAT 3´

The following PCR thermocycling regime was used: 94 °C for 2 min, followed by 40 cycles of 94 °C for 30 s, 52 °C for 50 s and 72 °C for 40 s, with a final elongation cycle of 72 °C for 10 min. Strong amplicons were subjected to the DNA barcoding sequencing protocol described by (Hebert et al*.*^60^). The resultant clean bi-directional sequences were trimmed to 497 bp (the maximum conserved length all samples).

### *RbcL* DNA barcoding of pollen loads by 454 pyrosequencing

Tagged *rbcL* primers were used to differentiate 454 sequences of pollen loads originating from different source bees as described previously by Meyer et al.^62^. Here, initial PCR primers are 5´-tagged with short nucleotide sequences (tags) in such a way that a unique tagged primer combination can be applied to each specific DNA template source coming from individual insect.

PCR amplicons from pollen loads of each bee were bulked by mixture (10 µl from each product) and purified using the PCR purification Qiagen Kit (Qiagen UK) according to manufacturers’ instructions. The resultant 454 sequencing templates comprised 14.5 µg of cleaned amplification product in total in volumes of approximately 100 µl (at 145 ng/µl as determined by nanodrop) (Thermo scientific-NanoDrop 1000). These samples were sent to Inqaba Biotechnical Industries (Pty) Ltd (South Africa) for 454 sequencing. The protocol used for 454 GS FLX titanium-based sequencing as described by Hawkins et al*.*^63^.

### Assembly of rbcL pollen profiles

The rbcL sequences were searched within the BOLD Systems V4 database using a Python script (available as freeware at: https://github.com/se-srl/bumblebee). For each RbcL sequence, the script used a curl command to retrieve responses from the BOLD Systems web page. This output was then parsed and plant identifiers with the maximum similarity are considered a match. The genus and species of each result was saved. When two or more query sequences uniquely matched to different reference barcodes of the same species, counts of both variants were amalgamated. Number of matches to each species were assembled onto an Excel spreadsheet. The analytical approach taken sought to emphasise comparison of profiles between individual bees. For this reason, counts representing matches to each pollen species were converted into percentages for that bee. In this way we sought to avoid skewing in favour of individuals with high count yields. Similarly, any bees with less than 500 total counts were removed from analysis to reduce skewing due to sample size. The resulting raw profiles contained percentage matches for all pollen species to all reference plant species. Two arbitrary thresholds were applied. The first (raw profiles), removed sequence counts comprising less than 1% of those present as these were considered as potential sources of contamination. For most analyses, a much higher threshold of 5% was imposed on individual bee profiles. This was intended to reduce the presence of externally foraged pollen and ensure that all pollen types present represented only the main preferences exhibited by individual bees. By definition, this action greatly reduced diversity of pollen types recovered from individual bees and meant that profiles would bias against incidental, occasional or external foraging events.

All statistical analyses and all NMDS analyses were performed using PAST software package^64^.

### Supplementary Information

Supplementary Information.

## Data Availability

Data generated in the current study are available from the corresponding author on request. All barcode sequences are freely available from the European Nucleotide Archive (ENA) (https://www.ebi.ac.uk/ena) under project accession number PRJEB58796.

## References

[1] Tilman D, Lehman C (2001). Human-caused environmental change: Impacts on plant diversity and evolution. Proc. Natl. Acad. Sci. USA.

[2] Potts SG, Biesmeijer JC, Kremen C, Neumann P, Schweiger O, Kunin WE (2010). Global pollinator declines: Trends, impacts and drivers. Trends Ecol. Evol..

[3] Grab H, Branstetter MG, Amon N, Urban-Mead KR, Park MG, Gibbs J, Blitzer EJ, Poveda K, Loeb G, Danforth BN (2019). Agriculturally dominated landscapes reduce bee phylogenetic diversity and pollination services. Science.

[4] Powney GD, Carvell C, Edwards M, Morris RKA, Roy HE, Woodcock BA, Isaac NJB (2020). Widespread losses of pollinating insects in Britain. Nat. Commun..

[5] Gallai N, Salles JM, Settele J, Vaissiere BE (2009). Economic valuation of the vulnerability of world agriculture confronted with pollinator decline. Ecol. Econ..

[6] Tscheulin T, Neokosmidis L, Petanidou T, Settele J (2011). Influence of landscape context on the abundance and diversity of bees in Mediterranean olive groves. Bull. Entomol. Res..

[7] Hardin G (1960). Competitive exclusion principle. Science.

[8] Holt RD, Grover J, Tilman D (1994). Simple rules for interspecific dominance in systems with exploitative and apparent competition. Am. Nat..

[9] Ashman TL, Knight TM, Steets JA, Amarasekare P, Burd M, Campbell DR, Dudash DR, Johnston MO, Mazer SJ, Mitchell RJ, Morgan MT, Wilson WG (2004). Pollen limitation of plant reproduction: Ecological and evolutionary causes and consequences. Ecology.

[10] Van der Kooi CJ, Pen I, Staal M, Stavenga DG, Elzenga JTM (2016). Competition for pollinators and intra-communal spectral dissimilarity of flowers. Plant Biol..

[11] Mallinger RE, Prasifka JR (2017). Bee visitation rates to cultivated sunflowers increase with the amount and accessibility of nectar sugars. J. Appl. Entomol..

[12] Ellis JS, Knight ME, Carvell C, Goulson D (2006). Cryptic species identification: A simple diagnostic tool for discriminating between two problematic bumblebee species. Mol. Ecol. Notes.

[13] Williams P (2007). The distribution of bumblebee colour patterns worldwide: Possible significance for thermoregulation, crypsis and warning mimicry. J. Linn. Soc. Bot..

[14] Williams PH, An JD, Brown MJF, Carolan JC, Goulson D, Huang JX, Ito M (2012). Cryptic bumblebee species: Consequences for conservation and the trade in greenhouse pollinators. PLoS One.

[15] Steen R (2017). Diel activity, frequency and visit duration of pollinators in focal plants: In situ automatic camera monitoring and data processing. Methods Ecol. Evol..

[16] Wolf S, Rohde M, Moritz RFA (2010). The reliability of morphological traits in the differentiation of Bombus terrestris and B. lucorum (Hymenoptera: Apidae). Apidologie.

[17] Scriven JJ, Woodall LC, Tinsley MC, Knight MIE, Williams PH, Carolan JC, Brown MJF, Goulson D (2015). Revealing the hidden niches of cryptic bumblebees in Great Britain: Implications for conservation. Biol. Conserv..

[18] Fussell M, Corbet SA (1991). Foraging bumble bees and honey bees in farmland: A case study. J. Apic. Res..

[19] Stout JC, Allen JA, Goulson D (2000). Nectar robbing, forager efficiency and seed set: Bumblebees foraging on the self-incompatible plant Linaria vulgaris (Scophulariaceae). Acta Oecol..

[20] Smithson A (2002). The consequences of rewardlessness in orchids: Reward-supplementation experiments with Anacamptis morio (Orchidaceae). AM. J. Bot..

[21] Stanley DA, Knight ME, Stout JC (2013). Ecological variation in response to mass-flowering oilseed rape and surrounding landscape composition by members of a cryptic bumblebee complex. PLoS One.

[22] Scriven JJ, Whitehorn PR, Goulson D, Tinsley MC (2016). Bergmann’s body size rule operates in facultatively endothermic insects: evidence from a complex of cryptic bumblebee species. PLoS One.

[23] Eckerter PW, Albus L, Natarajan S, Albrecht M, Ammann L, Gobet E, Herzog F, Tinner W, Entling MH (2020). Using temporally resolved floral resource maps to explain bumblebee colony performance in agricultural landscapes. Agronomy.

[24] Barrios, B., Pena, S. R., Salas, A., Koptur, S. (2016). Butterflies visit more frequently, but bees are better pollinators: the importance of mouthpart dimensions in effective pollen removal and deposition. *AOB Plants* **8**, article number: plw001. 10.1093/aobpla/plw001 PMC4779295 26742956

[25] Bell KL, Burgess KS, Botsch JC, Dobbs EK, Read TD, Brosi BJ (2019). Quantitative and qualitative assessment of pollen DNA metabarcoding using constructed species mixtures. Mol. Ecol..

[26] Bell KL, Fowler J, Burgess KS, Dobbs EK, Gruenewald D, Lawley B, Morozumi C, Brosi BJ (2017). Applying pollen DNA metabarcoding to the study of plant-pollinator interactions. Appl. Plant Sci..

[27] Lucas A, Bodger O, Brosi BJ, Ford CR, Forman DW, Greig C, Hegarty MM, Neyland PJ, de Vere N (2018). Generalisation and specialisation in hoverfly (Syrphidae) grassland pollen transport networks revealed by DNA metabarcoding. J. Anim. Ecol..

[28] Potter C, de Vere N, Jones LE, Ford CR, Hegarty MJ, Hodder KH, Dias A, Franklin EL (2019). Pollen metabarcoding reveals broad and species-specific resource use by urban bees. PeerJ.

[29] Pornon A, Andalo C, Burrus M, Escaravage N (2017). DNA metabarcoding data unveils invisible pollination networks. Sci. Rep..

[30] Richardson RT, Lin CH, Sponsler DB, Quijia JO, Goodell K, Johnson RM (2015). Applications of ITS2 metabarcoding to determine the provenance of pollen collected by honey bees in an agroecosystem. Appl. Plant Sci..

[31] Klecka J, Mikat M, Kolouskova P, Hadrava J, Straka J (2022). Individual-level specialisation and interspecific resource partitioning in bees revealed by pollen DNA metabarcoding. PeerJ.

[32] Fernandes K, Prendergast K, Bateman PW, Saunders BJ, Gibberd M, Bunce M, Nevill P (2022). DNA metabarcoding identifies urban foraging patterns of oligolectic and polylactic cavity-nesting bees. Oecologia.

[33] Villalta I, Ledet R, Baude M, Genaud D, Bouget C, Cornillon M, Moreau S, Courtial B, Lopez-Vaamonde C (2021). A DNA barcode-based survey of wild urban bees in the Loire Valley. France. Sci. Rep..

[34] Goodwin SG (1995). Seasonal phenology and abundance of early-, mid and long-season bumble bees in southern England, 1985–1989. J. Apic. Res..

[35] Walther-Hellwig K, Frankl R (2000). Foraging habitats and foraging distances of bumblebees, Bombus spp. (Hym., apidae), in an agricultural landscape. J. Appl. Entomol..

[36] Redhead JW, Dreier S, Bourke AFG, Heard MS, Jordan WC, Summer S, Wang JL, Carvell C (2016). Effects of habitat composition and landscape structure on worker foraging distances of five bumblebee species. Ecol. Appl..

[37] Knight ME, Martin AP, Bishop S, Osborne JL, Hale RJ, Sanderson A, Goulson D (2005). An interspecific comparison of foraging range and nest density of four bumblebee (Bombus) species. Mol. Ecol..

[38] Spaethe J, Weidenmuller A (2002). Size variation and foraging rate in bumblebees (Bombus terrestris). Insectes Sociaux.

[39] Muller H (2012). Individual consistency in foraging behaviour and response to predator threat in the bumblebee Bombus terrestris (Hymenoptera: Apidae). Entomol. Gen..

[40] Raine NE, Ings TC, Ramos-Rodriguez O, Chittka L (2006). Intercolony variation in learning performance of a wild British bumblebee population (Hymenoptera: Apidae: Bombus terrestris audax). Entomol. Gen..

[41] Kendall LK, Mola JM, Portman ZM, Cariveau DP, Smith HG, Bartomeus I (2022). The potential and realized foraging movements of bees are differentially determined by body size and sociality. Ecology.

[42] Westphal C, Steffan-Dewenter I, Tscharntke T (2006). Bumblebees experience landscapes at different spatial scales: possible implications for coexistence. Oecologia.

[43] Iwasaki JM, Dickinson KJM, Barratt BIP, Mercer AR, Jowett TWD, Lord JM (2018). Floral usage partitioning and competition between social (Apis mellifera, Bombus terrestris) and solitary bees in New Zealand: Niche partitioning via floral preferences?. Austral Ecol..

[44] Nishikawa Y, Shimamura T, Kudo G, Yabe K (2019). Habitat use and floral resource partitioning of native and alien bumblebees in the coastal grassland-rural landscape. J. Insect Conserv..

[45] Namin SM, Kim MJ, Son M, Jung C (2022). Honey DNA metabarcoding revealed foraging resource partitioning between Korean native and introduced honey bees (Hymenoptera: Apidae). Sci. Rep..

[46] Ronca S, Allainguillaume J, Ford CS, Warren J, Wilkinson MJ (2017). GM risk assessment: Pollen carriage from *Brassica napus* to *B. rapa* varies widely between pollinators. Basic Appl. Ecol..

[47] Arbulo N, Santos E, Salvarrey S, Invernizzim C (2011). Proboscis length and resource utilization in two Uruguayan bumblebees: *Bombus atratus* Franklin and *Bombus bellicosus* Smith (Hymenoptera: Apidae). Neotrop. Entomol..

[48] Marchand P, Harmon-Threatt AN, Chapela I (2015). Testing models of bee foraging through the analysis of pollen loads and floral density data. Ecol Model.

[49] Sponsler D, Kallnik KF, Classen A, Maihoff AF, Sieger J, Steffan-Dewenter I (2022). Floral preferences of mountain bumble bees are constrained by functional traits but flexible through elevation and season. OIKOS.

[50] Wilson RS, Keller A, Shapcott A, Leonhardt SD, Sickel W, Hardwick JL, Heard TA, Kaluza BF, Wallace HM (2021). Many small rather than few large sources identified in long-term bee pollen diets in agroecosystems. Agric. Ecosyst. Environ..

[51] Szigeti V, Korosi A, Harnos A, Nagy J, Kis J (2016). Measuring floral resource availability for insect pollinators in temperate grasslands: A review. Ecol. Entomol..

[52] Goulnik J, Plantureux S, Dajoz I, Michelot-Antalik A (2021). Using matching traits to study the impacts of land-use intensification on plant-pollinator interaction in European grasslands: A review. Insects.

[53] Ouvrard P, Transon J, Jacquemart A-L (2018). Flower-strip agri-environment schemes provide diverse and valuable summer flower resources for pollinating insects. Biodivers. Conserv..

[54] Bansch S, Tscharntke T, Wunschiers R, Netter L, Brenig B, Gabriel D, Westphal C (2020). Using ITS2 metabarcoding and microscopy to analyse shifts in pollen diets of honey bees and bumble bees along a mass-flowering crop gradient. Mol. Ecol..

[55] Vazquez DP, Morris WF, Jordano P (2005). Interaction frequency as a surrogate for the total effects of animal mutualists on plants. Ecol. Lett..

[56] Lunt, I. D. (1994). Variation in flower production of nine grassland species with time since fire, and implications for grassland management and restoration in *Pac. Conserv. Biol.* **1**: 359–366

[57] Stace, C. A. (2019). New Flora of the British Isles, 4th Edition. C&M Floristics, Suffolk UK. pp. 1266. ISBN: 0521589355.

[58] Falk S, Foster G, Comont R, Conroy J, Bostock H, Salisbury A, Kilbey D, Bennett J, Smith B (2019). Evaluating the ability of citizen scientists to identify bumblebee (Bombus) species. PLos One.

[59] Memmott J (1999). The structure of a plant-pollinator food web. Ecol. Lett..

[60] Hebert P, Penton E, Burns J, Janzen D, Hallwachs W (2004). Ten species in one: DNA barcoding reveals cryptic species in the neotropical skipper butterfly Astraptes fulgerator. Proc. Natl. Acad. Sci. USA.

[61] Kress WJ, Erickson DL (2007). A two-locus global DNA barcode for land plants: the coding rbcL gene complements the non-coding trnH-psbA spacer region. PLoS One.

[62] Meyer M, Stenzel U, Hofreiter M (2008). Parallel tagged sequencing on the 454 platform. Nat. Protoc..

[63] Hawkins J, de Vere N, Griffith A, Ford CR, Allainguillaume J, Hegarty MJ, Baillie L, Adams-Groom B (2015). Using DNA metabarcoding to identify the floral composition of honey: A new tool for investigating honey bee foraging preferences. PLoS One.

[64] Hammer Ø, Harper DAT, Ryan PD (2001). PAST: Paleontological Statistics software package for education and data analysis. Palaeontologia Electronica.

