# Author Correction: The value of twinned pollinator-pollen metabarcoding: bumblebee pollination service is weakly partitioned within a UK grassland community

**DOI:** 10.1038/s41598-023-47587-7

**Published:** 2023-11-24

**Authors:** Sandra Ronca, Caroline S. Ford, Joël Allanguillaume, Claudia Szabo, Richard Kipling, Mike J. Wilkinson

**Affiliations:** 1 Department of Life Sciences, Aberystwyth University, Aberystwyth, SY23 3DA UK; 2 Wales Veterinary Science Centre, Y Buarth, Aberystwyth, SY23 1ND Ceredigion UK; 3 Department of Biological, Biomedical and Analytical Sciences, University of the West of England, Coldharbour Lane, Bristol, BS16 1QY UK; 4 School of Computer Science, The University of Adelaide, Adelaide, SA 5005 Australia; 5 The Sustainable Food Trust, 38 Richmond Street, Totterdown, Bristol, BS3 4TQ UK

Correction to: *Scientific Reports* 10.1038/s41598-023-44822-z, published online 21 October 2023

The original version of this Article contained an error in the spelling of the author Joël Allanguillaume, which was incorrectly given as Joël Allanguillame.

The original Article has been corrected.

