# Supplementary material for: The value of twinned pollinator-pollen metabarcoding: bumblebee pollination service is weakly partitioned within a UK grassland community
Source: Sci Rep. 2023 Oct 21;13:18016. doi: 10.1038/s41598-023-44822-z (PMC10590402; doi:10.1038/s41598-023-44822-z)
Supplement: Supplementary file 1 — Supplementary Information. [file 41598_2023_44822_MOESM1_ESM.pdf]

Figure S1. Neighbour Joining tree showing variation in Cytochrome Oxidase 1 (CO1) reference barcode sequences for eight *Bombus* species retrieved from the BOLD Systems V4 database and query CO1 sequences from bees captured at the study sites.

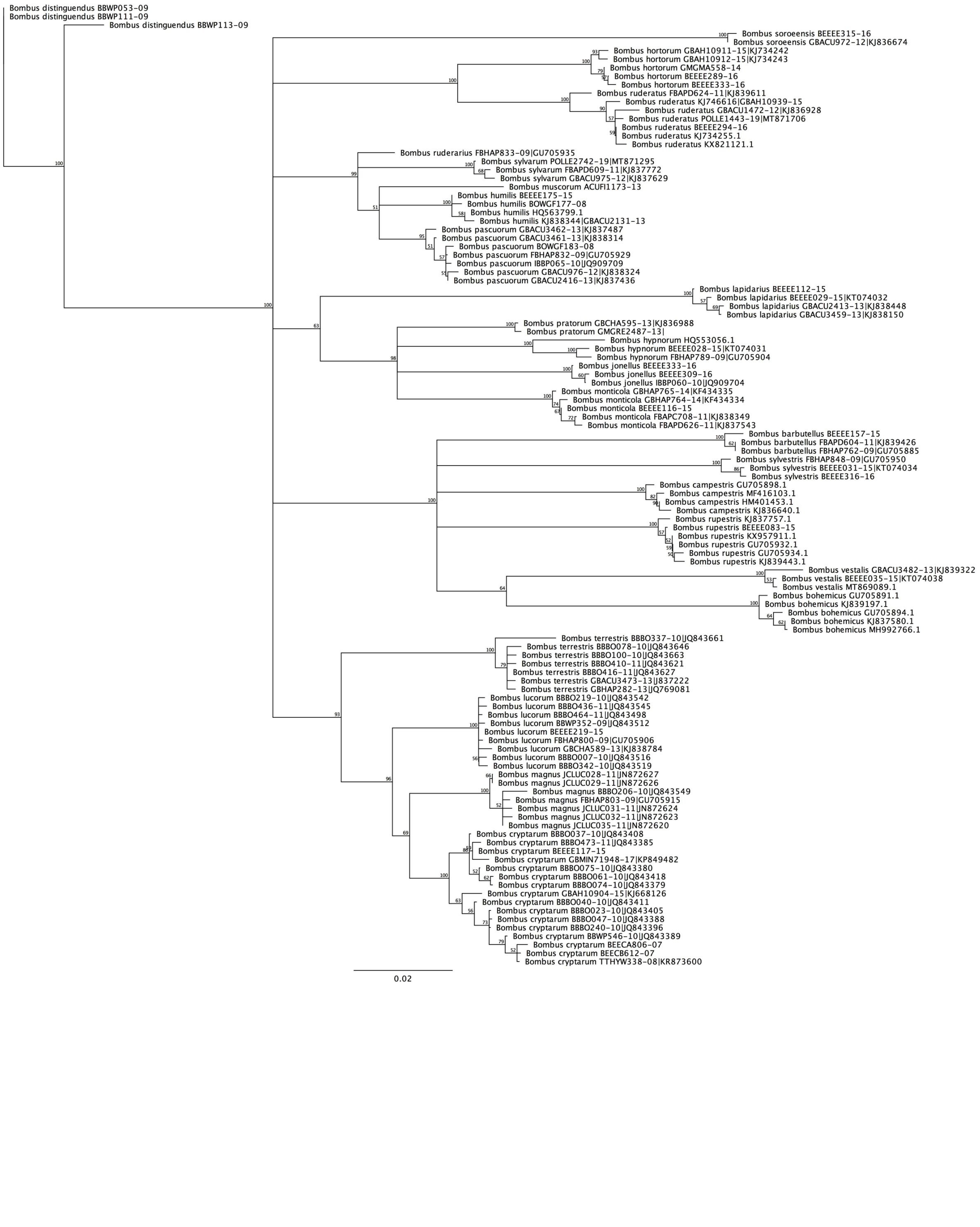

Figure S2. Histogram plots of the overall relative percentage of pollen from each plant species carried by bees captured in (A) RF; (B) RG and (C) RGYH). Cumulative amount of pollen from each plant species (the sum of the % carried across all 272 individual bees) from (D) RF; (E) RG; (F) RGYH.

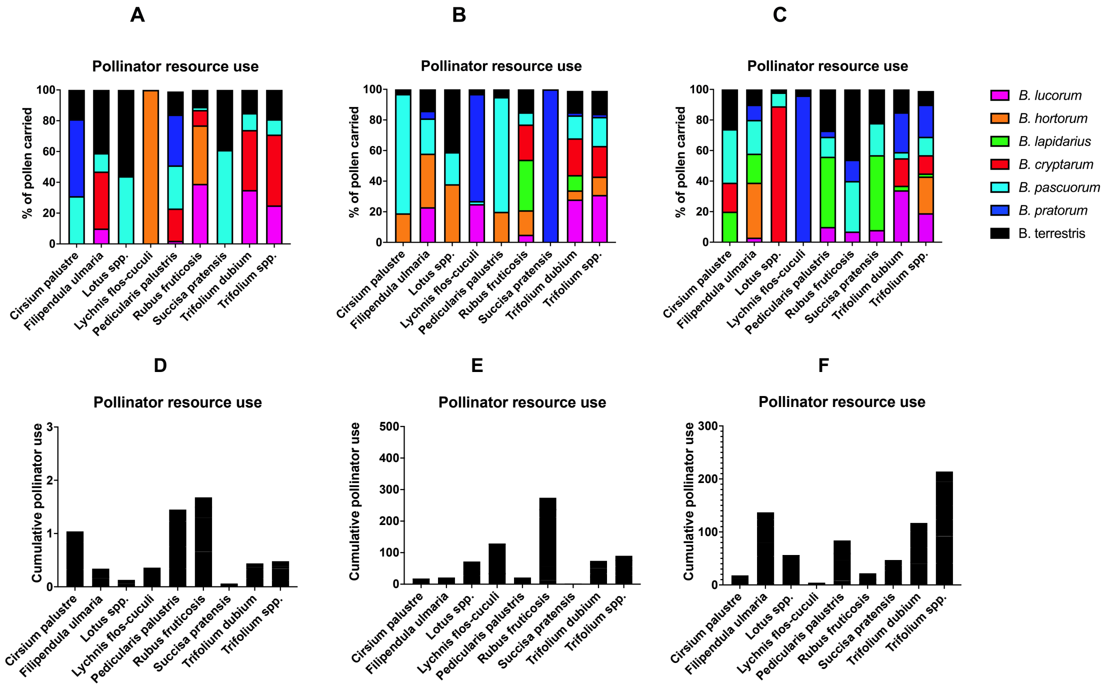

Table S1. Heat map showing the relative abundance of flowering heads from insect-pollinated plant species recorded across the three Rhos pasture study sites, where represents no flowers; 1-49 flowers; 50-99 flowers; 100-999 flowers; 1000+ flowers per transect

**Plant Species**	**June 16-18**	**July 8 -13**	**July 24-27**	**August 5-7**	**August 24-25**
Cirsium palustre	8	15		3	2
Pilosella officinarum	10				
Myosotis scorpioides	33	10			
Myosotis arvensis	3				
Cardamine pratensis	20				
Stellaria holostea	10	19	11	1	5
Stellaria uliginosa	44	15	1	4	
Stellaria graminae	6	10		4	
Lychnis flos-cuculi	39				
Erica tetralix	3	11	42	200	33
Trifolium dubium	175	18	11		
Trifolium pratense	10	24	3	3	
Trifolium repens	60	83	14	10	
Lathyrus pratensis	8	23	4	0	
Lotus pedunculatus	6	1948	916	232	23
Lotus corniculatus	54			5	
Prunella vulgaris	7	26	28	6	
Epilobium montanum	4				
Dactylorhiza maculata	22	15			
Dactylorhiza praetermissa	4				
Ranunculus acris	205	65	5		
Ranunculus flammula	185	269	58	104	38
Ranunculus repens	71	2	1	1	
Potentilla erecta	109	333	40	198	52
Galium palustre	22	2287	126	13	
Galium saxatile	46	12			
Veronica serpyllifolia	14				
Euphrasia nemorosa	15	211		75	40
Pedicularis palustris	20				
Pedicularis sylvatica	23	8			
Rhinanthus minor	212	43	27		
Conopodium majus		1006	36	278	16
Achillea ptarmica		7	26	14	
Senecio jacobaea/aquaticus		23	137	70	50
Epilobium palustre		2	4	11	
Anagallis tenella		4			
Rubus fruticosus		22	7	4	
Veronica scutellata		33		3	
Hypochoeris radicata			1		
Leontodon autumnalis			1	6	2
Scutellaria minor			4		
Filipendula ulmaria			11	7	
Angelica sylvestris				42	192
Centaurea nigra				7	
Drosera rotundifolia				1	
Calluna vulgaris				6	
Succisa pratensis					110
Total Flowering Heads	**1448**	**6544**	**1514**	**1308**	**563**

Table S2

A. Table showing the number of individual bees for each bee species c

Bee species	*Digitalis purpurea*	*Trifolium dubium*
B. lucorum	3	14
B. hortorum	9	5
B. lapidarius	3	7
B. cryptarum	5	11
B. pascuorum	16	46
B. pratorum	1	5
B. terrestris	14	49

B. Table showing the number of individual bees for each bee species c

Bee species	*Digitalis purpurea*	*Trifolium dubium*
B. lucorum	3	12
B. hortorum	6	1
B. lapidarius	0	4
B. cryptarum	4	7
B. pascuorum	3	27
B. pratorum	0	2
B. terrestris	8	30

Table S3. Overall pollen carriage profile (percentage of each plant species barcode) across all sites and collection periods.

Bee species	*Pedicularis palustris*	*Trifolium*	*Filipendula ulmaria*	*Centaurea nigra*	*Rubus fruticosus*	*Succisa pratensis*	*Cirsium palustre*	*Achillea*	*Calluna vulgaris*	*Lotus*	*Valeriana officinalis*	*Lychnis flos-cuculi*	Others
B. cryptarum	21	17	14	12	10	10	6	3	2	1	1	0	3
B. hortorum	20	18	14	12	10	10	6	3	1	1	1	0	3
B. lapidarius	21	17	14	12	10	10	6	3	2	1	1	0	3
B. lucorum	20	18	14	12	10	10	6	3	1	1	1	0	4
B. pascuorum	17	17	12	7	9	7	8	2	1	1	0	7	12
B. pratorum	21	18	14	12	10	10	6	3	2	1	1	0	3
B. terrestris	20	18	14	11	10	10	6	3	2	1	1	0	4

Table S4

A. Presence of different types of pollen as revealed by *rbcL* metabarcoding that was recovered from individual bees (expressed as a percentage of all pollen from the bee) recorded across three study sites

Bee identifier	Bee species	Site/Collection date	*Digitalis purpurea*	*Trifolium dubium*	*Galium palustre*	*Ajuga reptans*	*Trifolium species*	*Lychnis flos-cuculi*	*Pedicularis palustris*	*Stachys betonica*	*Rubus fruticosus*	*Cirsium palustre*	*Erica tetralix*	*Calluna vulgaris*	*Filipendula ulmaria*	*Lotus species*	*Valeriana officinalis*	[illegible]
		Present in study site	+	+	+	+	+	+	+	+	+	+	+	+	+	+	+	+
		Present in 10km region	+	+	+	+	+	+	+	+	+	+	+	+	+	+	+	+
21	*B. lucorum*	RG 7/08/09	6	6	4	0	11	0	1	0	65	0	0	0	0	0	0	1
33	*B. lucorum*	RGHY 5/08/2009	0	6	0	0	8	0	28	0	5	1	11	7	3	0	2	0
71	*B. lucorum*	RGHY 26/08/2009	0	6	0	0	5	0	0	1	0	0	1	22	16	1	1	0
115	*B. lucorum*	RG 13/07/09	0	48	0	0	41	0	0	0	5	0	0	0	0	0	0	0
132	*B. lucorum*	RF 8/07/09	0	23	0	0	18	0	0	0	45	0	0	0	2	0	1	0
134	*B. lucorum*	RF 8/07/09	0	6	0	0	5	0	0	0	80	0	0	0	0	0	1	0
144	*B. lucorum*	RGHY 18/06/09	15	30	0	1	30	0	0	0	1	0	0	0	0	0	0	0
146	*B. lucorum*	RGHY 18/06/09	0	46	0	0	48	0	0	0	0	0	0	0	0	0	0	0
157	*B. lucorum*	RG 15/06/09	0	33	0	1	41	0	0	0	0	0	0	1	0	0	0	0
160	*B. lucorum*	RG 15/06/09	0	0	0	4	0	0	0	0	0	0	0	0	0	0	0	0
162	*B. lucorum*	RG 15/06/09	0	3	0	0	0	81	0	0	2	0	0	0	0	0	0	0
164	*B. lucorum*	RG 15/06/09	5	0	0	26	29	0	0	0	0	0	0	0	26	0	0	0
166	*B. lucorum*	RG 15/06/09	0	0	0	0	0	94	0	0	0	0	0	0	0	0	0	0
167	*B. lucorum*	RG 15/06/09	0	26	0	3	30	0	0	0	0	0	0	0	0	1	0	0
194	*B. lucorum*	RF 16/06/2009	0	1	0	2	0	0	7	0	1	0	0	0	7	1	0	0
220	*B. lucorum*	RGHY 5/08/08	0	48	0	0	47	0	0	0	0	0	0	0	0	0	0	0
286	*B. lucorum*	RGHY 22/08/08	0	33	0	0	46	0	0	0	0	0	0	0	5	0	0	0
1	*B. hortorum*	RG 27/07/2009	33	3	48	2	4	1	1	1	0	0	0	0	0	0	0	0
2	*B. hortorum*	RG 27/07/2009	2	0	0	2	0	0	0	22	53	0	0	0	0	0	0	0
3	*B. hortorum*	RG 27/07/2009	12	2	3	2	4	0	0	3	46	1	0	0	0	0	0	0
15	*B. hortorum*	RG 7/08/09	41	0	3	5	1	0	1	22	2	1	0	0	1	0	0	0
17	*B. hortorum*	RG 7/08/09	1	0	0	2	0	0	27	4	16	24	0	0	1	0	0	0
18	*B. hortorum*	RG 7/08/09	1	0	0	1	22	0	0	10	29	0	0	0	24	0	0	0
19	*B. hortorum*	RG 7/08/09	0	0	0	0	25	0	0	0	44	0	0	0	26	0	0	0
20	*B. hortorum*	RG 7/08/09	86	0	2	6	0	0	0	1	1	1	0	0	0	1	0	0
32	*B. hortorum*	RGHY 5/08/2009	0	1	0	0	46	0	3	0	0	0	0	0	37	0	1	0
106	*B. hortorum*	RGHY 10/07/09	0	0	0	0	45	0	0	0	0	0	0	0	48	0	0	0
107	*B. hortorum*	RGHY 10/07/09	0	0	0	0	47	0	0	0	0	0	0	0	47	0	0	0
113	*B. hortorum*	RG 13/07/09	11	0	8	1	4	0	1	9	48	0	0	0	7	0	0	0
114	*B. hortorum*	RG 13/07/09	1	0	0	1	0	0	0	21	56	0	0	0	0	0	0	0
240	*B. hortorum*	RF 24/08/08	26	0	0	1	0	19	0	7	34	0	0	0	1	0	0	0
257	*B. hortorum*	RG 15/08/08	0	1	0	0	2	0	0	0	2	0	0	0	0	83	0	0
258	*B. hortorum*	RG 15/08/08	0	0	0	0	0	0	3	0	48	2	0	0	1	33	0	0
259	*B. hortorum*	RG 15/08/08	0	0	0	0	1	0	5	0	1	3	0	0	0	81	0	0
260	*B. hortorum*	RG 15/08/08	0	34	0	0	41	0	0	0	0	0	0	0	0	14	0	0
48	*B. lapidarius*	RGHY 5/08/2009	1	1	5	0	0	0	29	0	1	0	0	0	8	0	7	0
49	*B. lapidarius*	RGHY 5/08/2009	0	7	1	0	0	0	38	0	3	1	0	0	5	1	0	0

[illegible]

B. Presence of different types of pollen as revealed by rbcL barcoding that was recovered from individual bees (all pollen types above 5% threshold of pollen from the bee) recorded across three study sites

[illegible]

Fig S6. Comparison of pollen carriage profiles of bee species. Data are compared by Permanova following data transformation of 454 count frequencies of individual bees into ternary format, where counts <5% score 0; Counts 5%<x<50% score 1 and counts >50% score 2.
N-MDS plots of significant species pairings and associated stress test results shown below

Permanova

PERMANOVA

Permutation N:	9999
Total sum of squares:	1.651
Within-group sum of squares:	1.433
F:	6.665
p (same):	0.0001

Pairwise comparisons

Bonferroni-corrected p values

	B. lucorum	B. hortorum	B. lapidarius	B. cryptarum	B. pascuorum	B. pratorum	B. terrestris
B. lucorum		0.0042	0.0021	1	0.0021	0.0021	0.0882
B. hortorum	0.0042		0.0042	0.2331	0.0021	0.0021	0.0189
B. lapidarius	0.0021	0.0042		0.0021	0.0021	0.0021	0.0063
B. cryptarum	1	0.2331	0.0021		0.0504	0.0336	1
B. pascuorum	0.0021	0.0021	0.0021	0.0504		0.0021	0.6174
B. pratorum	0.0021	0.0021	0.0021	0.0336	0.0021		0.0042
B. terrestris	0.0882	0.0189	0.0063	1	0.6174	0.0042	

N-MDS plots of significant species pairings with stress values <0.2

Species pairing	N-MDS plot	Stress value	Species pairing	N-MDS plot	Stress value
B. lucorum-B.hortorum	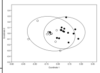	0.268	*B. lapidarius-B.cryptarum*	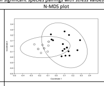	0.241
B. lucorum-B.lapidarius	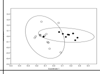	0.216	*B.lapidarius-B.pascuorum*	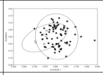	0.265
B. lucorum-B. pascuorum	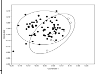	0.265	*B. lapidarius- B.pratorum*	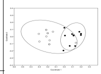	0.206
B. lucorum-B. pratorum	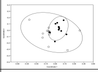	0.238	*B. lapidarius-B.terrestris*	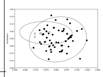	0.273
B. hortorum- B. lapidarius	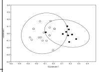	0.238	*B.cryptorum-B.pratorum*	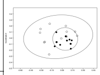	0.246
B. hortorum- B. pascuorum	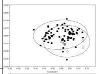	0.281	*B. pascuorum-B. pratorum*	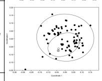	0.283
B. hortorum- B. pratorum	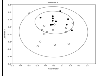	0.284	*B. pratorum-B.terrestris*	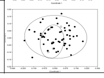	0.291
B.hortorum-B. terrestris	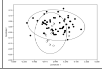	0.297			

Fig S7. Comparison of pollen carriage profiles of bee species using quantitative data over a 5% threshold.
The 454 count frequencies (above 5% threshold) are compared by Permanova.

Permanova

PERMANOVA

Permutation *N*:	9999
Total sum of squares:	7.756E05
Within-group sum of squares:	6.905E05
F:	5.401
p (same):	0.0001

Similarity index: Euclidean

Permutation N: 9999

Pairwise comparisons

Bonferroni-corrected p values

	B. lucorum	B. hortorum	B. lapidariu:	B. cryptarur	B. pascuoru	B. pratorum	B. terrestris
3. lucorum		0.1743	0.0042	1	0.0042	0.0903	0.1722
3. hortorum	0.1743		0.0042	1	0.0315	0.0021	0.2982
3. lapidariu:	0.0042	0.0042		0.042	0.3801	0.0063	0.6762
3. cryptarur	1	1	0.042		0.3381	0.0063	1
3. pascuoru	0.0042	0.0315	0.3801	0.3381		0.0021	0.4452
3. pratorum	0.0903	0.0021	0.0063	0.0063	0.0021		0.0021
3. terrestris	0.1722	0.2982	0.6762	1	0.4452	0.0021	

Fig S8. Comparison of pollen carriage profiles of bee species in A. RF; B. RG and C. RGHY using quantitative data over a 5% threshold.

The 454 count frequencies (above 5% threshold) are compared by Permanova.

A

Permanova

PERMANOVA

Permutation *N*:	9999
Total sum of squares:	1.156E05
Within-group sum of squares:	9.463E04
F:	2.035
p (same):	0.0107

Similarity index: Euclidean

Permutation N: 9999

Pairwise comparisons

Bonferroni-corrected p values

	B. lucorum	B. hortorum	B. cryptarur	B. pascuoru	B. pratorum	B. terrestris
B. lucorum		1	1	0.204	1	1
B. hortorum	1		1	1	1	1
B. cryptarur	1	1		0.042	1	1
B. pascuoru	0.204	1	0.042		1	0.3975
B. pratorum	1	1	1	1		1
B. terrestris	1	1	1	0.3975	1	

B

Permanova

PERMANOVA

Permutation *N*:	9999
Total sum of squares:	3.509E05
Within-group sum of squares:	2.722E05
F:	4.386
p (same):	0.0001

Similarity index: Euclidean

Permutation N: 9999

Pairwise comparisons

Bonferroni-corrected p values

	B. lucorum	B. hortorum	B. lapidarius	B. cryptarur	B. pascuoru	B. pratorum	B. terrestris
B. lucorum		0.0924	1	1	0.2436	1	0.6741
B. hortorum	0.0924		1	1	1	0.0021	1
B. lapidarius	1	1		1	1	1	1
B. cryptarur	1	1	1		1	0.336	1
B. pascuoru	0.2436	1	1	1		0.0021	0.8631
B. pratorum	1	0.0021	1	0.336	0.0021		0.0021
B. terrestris	0.6741	1	1	1	0.8631	0.0021	

C

Permanova

PERMANOVA

Permutation *N*:	9999
Total sum of squares:	3.624E05
Within-group sum of squares:	3.08E05
F:	3.292
p (same):	0.0001

Similarity index: Euclidean

Permutation N: 9999

Pairwise comparisons

Bonferroni-corrected p values

	B. lucorum	B. hortorum	B. lapidarius	B. cryptarur	B. pascuoru	B. pratorum	B. terrestris
B. lucorum		0.2457	0.0021	1	0.0378	1	0.4872
B. hortorum	0.2457		0.0609	1	1	1	0.0693
B. lapidarius	0.0021	0.0609		0.063	0.0147	0.0126	1
B. cryptarur	1	1	0.063		0.5481	1	0.8127
B. pascuoru	0.0378	1	0.0147	0.5481		1	0.0063
B. pratorum	1	1	0.0126	1	1		1
B. terrestris	0.4872	0.0693	1	0.8127	0.0063	1	

Table S9. Comparison of capture rates of *B. cryptarum* and *B. lucorum*

Site/date	*Bombus cryptarum*	*Bombus lucorum*
RG 15/06/09	1	6
RF 16/06/2009	6	1
RGHY 18/06/09	1	2
RF 8/07/09	1	2
RGHY 10/07/09	1	0
RG 13/07/09	1	0
RG 27/07/2009	3	0
RGHY 5/08/08	0	2
RF 6/08/09	1	0
RG 7/08/09	1	1
RGHY 22/08/08	0	1
RGHY 27/08/2009	1	1
**Total**	17	16

Fisher's exact test, p (no association) = 0.037

Fig S10. Comparison of profiles of bees that carry the nine most common pollen species.

A. Comparison of pollinator profiles between plant species.

Permanova

PERMANOVA

**Permutation *N*:**	9999
**Total sum of squares:**	3.663E06
**Within-group sum of squares:**	3.038E06
***F*:**	0.6946
***p* (same):**	0.7568

Pairwise comparisons

Bonferroni-corrected p values

	Trifolium du	Trifolium pr	Lychnis flos	Pedicularis	Rubus frutic	Cirsium palu	Filipendula	Lotus spp.	Succisa pra
Trifolium du		1	1	1	1	1	1	1	1
Trifolium pr	1		1	1	1	1	1	1	1
Lychnis flos	1	1		1	1	1	1	1	1
Pedicularis	1	1	1		1	1	1	1	1
Rubus frutic	1	1	1	1		1	1	1	1
Cirsium palu	1	1	1	1	1		1	1	1
Filipendula	1	1	1	1	1	1		1	1
Lotus spp.	1	1	1	1	1	1	1		1
Succisa pra	1	1	1	1	1	1	1	1	

B. Comparison of pollinator profiles from different sites

Permanova

PERMANOVA

**Permutation *N*:**	9999
**Total sum of squares:**	4.981E06
**Within-group sum of squares:**	4.695E06
***F*:**	0.7309
***p* (same):**	0.6246

Pairwise comparisons

Bonferroni-corrected p values

	RF	RG	RGHY
**RF**		1	0.9138
**RG**	1		0.9417
**RGHY**	0.9138	0.9417	

Fig S11. Effect of season on profiles of bees that carry the nine most common pollen species.

Comparison of pollinator profiles between June, July, early August and late August.

Permanova for all periods

PERMANOVA

Permutation N:	9999
Total sum of squares:	2472
Within-group sum of squares:	2363
F:	3.52
p (same):	0.0001

Similarity index: Euclidean

Permutation N: 9999

Pairwise comparisons

Bonferroni-corrected p values

	June	July	E.August	L.August
une		0.2124	0.0006	0.0006
uly	0.2124		0.0588	0.0174
.August	0.0006	0.0588		1
.August	0.0006	0.0174	1	

Table S12. Plant survey and bee sampling dates at the three study sites, where RF= Rhos Fullbrook; RG= Rhos Glandenys and RGHY=Rhos Glyn yr Helyg

RF		RG		RGHY	
2008	2009	2008	2009	2008	2009
	16th June		15th June		18th June
	8th July		13th July		10th July
	24th July		27th July		-
5th August	6th August	4th August	7th August	5th August	5th August
24th August	24th August	15th August	25th August	22nd August	26th August

[illegible]

arrying >0.99% of pollen for each of the named plant species

Galuim palustre	*Ajuga reptans*	*Trifolium species*	*Lychnis flos-cuculi*
1	6	13	2
5	9	11	1
4	0	7	0
2	3	10	0
3	13	65	3
0	7	6	12
4	7	49	4

arrying >5% of pollen for each of the named plant species

Galuim palustre	*Ajuga reptans*	*Trifolium species*	*Lychnis flos-cuculi*
0	1	13	2
2	2	6	1
1	0	4	0
1	1	8	0
0	5	50	2
0	3	3	11
1	3	32	2

Pedicularis palustris	*Stachys betonica*	*Rubus fruticosis*	*Cirsium palustre*	*Erica tetralix*
3	1	8	1	2
5	8	12	6	0
11	0	4	5	0
9	0	10	3	1
73	15	43	57	2
2	0	3	2	0
45	1	40	25	2

Pedicularis palustris	*Stachys betonica*	*Rubus fruticosis*	*Cirsium palustre*	*Erica tetralix*
2	0	5	0	1
2	6	9	1	0
10	0	1	2	0
5	0	5	1	1
56	7	29	46	0
2	0	2	1	0
36	0	31	16	1

Calluna vulgaris	*ipendula ulmar*	*Lotus species*	*Valeriana officinalis*
3	6	3	4
0	8	4	0
0	11	1	1
2	7	2	0
7	67	35	1
2	4	0	1
4	47	22	16

Calluna vulgaris	*ipendula ulmar*	*Lotus species*	*Valeriana officinalis*
2	4	0	0
0	6	4	0
0	11	0	1
2	3	1	0
2	47	21	0
0	2	0	0
3	34	17	5

Polygala vulgaris	*Rhinanthus minor*	*Prunella vulgaris*	*lea ptar.*	*OTU 1*
1	3	0	1	0
0	0	3	0	2
0	0	0	0	0
0	3	0	0	0
0	6	5	1	5
1	8	0	0	0
0	3	1	0	0

Polygala vulgaris	*Rhinanthus minor*	*Prunella vulgaris*	*lea ptar.*	*OTU 1*
0	3	0	0	0
0	0	2	3	0
0	0	0	0	0
0	1	0	0	1
0	6	1	5	1
1	8	0	0	0
0	1	1	0	4

Plant species from the study site

Stachys species	*:dicularis sylvat*	*Ulex species*	*'ardamine pratensi*
0	4	3	1
8	1	0	2
0	1	2	2
0	1	0	1
8	6	7	2
0	2	0	1
0	5	3	5

Stachys species	*:dicularis sylvat*	*Ulex species*	*'ardamine pratensi*
1	0	1	0
0	0	0	0
0	1	11	0
0	0	0	0
4	1	12	2
0	1	0	0
1	2	23	0

Plant species/Genus/OTU

es

Centaurea nigra	*Lathyrus pratensis*	*Succisa pratensis*	*hoxanthum odorat*
1	1	2	0
1	1	0	0
11	0	7	0
0	0	0	0
20	4	40	0
0	0	1	1
27	0	13	0

Centaurea nigra	*Lathyrus pratensis*	*Succisa pratensis*	*hoxanthum odorat*
2	1	0	0
0	0	0	0
6	0	0	0
0	3	0	1
22	0	1	0
1	0	0	0
13	0	0	0

Potentilla erecta	*Euphrasia nemorosa*	*Trifolium pratense/repens*
1	1	0
0	0	0
1	0	0
5	1	0
1	0	1
0	0	0
0	0	0

Potentilla erecta	*Euphrasia nemorosa*	*Trifolium pratense/repens*
1	0	1
0	0	0
0	0	0
3	0	3
1	1	0
0	0	0
2	0	0

larthecium ossifragur	*Ranunculus species*	*Veronica species*	*Scutellaria minor*	*ntilla spe*
0	4	0	0	2
0	0	0	0	0
0	0	0	0	0
1	3	0	0	6
0	2	1	1	0
0	2	0	0	0
0	4	0	0	0

larthecium ossifragur	*Ranunculus species*	*Veronica species*	*Scutellaria minor*	*ntilla spe*
0	1	0	1	0
0	0	0	0	0
1	0	0	0	0
0	0	0	1	0
0	0	1	0	1
0	1	0	0	0
0	0	0	2	2

OTU 2	*Potentilla palustris*	*Epilobium species*	*Veronica officinalis*
0	1	1	1
0	0	0	0
3	0	0	0
1	1	0	1
4	0	1	0
0	1	0	0
7	0	1	3

OTU 2	*Potentilla palustris*	*Epilobium species*	*Veronica officinalis*
0	0	0	1
0	0	0	2
0	0	0	2
1	0	0	3
1	0	0	21
0	1	0	2
0	0	1	11

Lotus corniculatus	*.eontodon autumnali:*	*Ranunculus species*	*ria serpy*
0	0	1	3
0	0	0	1
0	0	0	0
0	0	2	0
3	1	1	1
0	0	0	10
6	0	1	2

Lotus corniculatus	*.eontodon autumnali:*	*Ranunculus species*	*ria serpy*
0	0	1	3
0	0	0	1
0	0	0	0
0	0	2	0
3	1	1	1
0	0	0	10
6	0	1	2

OTU 3	*Angelica sylvestris*	*Apium inundatum*	*Alnus glutinosa*	*emilla sp*
0	1	0	1	3
0	0	0	3	5
0	0	0	0	1
0	0	0	1	2
0	1	1	10	15
0	0	0	0	0
1	0	0	5	16

OTU 3	*Angelica sylvestris*	*Apium inundatum*	*Alnus glutinosa*	*emilla sp*
0	1	0	1	3
0	0	0	3	5
0	0	0	0	1
0	0	0	1	2
0	1	1	10	15
0	0	0	0	0
1	0	0	5	16

OTU 4	*ostis spe*	*OTU 5*	*ostis vinealis/ can*	*hillea speci*
0	0	1	0	4
0	0	0	0	4
1	0	0	0	11
0	0	2	0	5
0	2	0	0	55
1	0	1	0	4
0	0	0	3	36

OTU 4	*ostis spe*	*OTU 5*	*ostis vinealis/ can*	*hillea speci*
0	0	1	0	4
0	0	0	0	4
1	0	0	0	11
0	0	2	0	5
0	2	0	0	55
1	0	1	0	4
0	0	0	3	36

es	OTU numbe	Species included
	1	*Mentha aquatica;Prunella vulgaris*
	2	*Filipendula ulmaria;Potentilla anse*
	3	*Chamerion angustifolium;Anagallis*
	4	*Populus nigra;Cardamine pratensis*
	5	*Viburnum opulus;Adoxa moschatel*

es	OTU numbe	Species included
	1	*Mentha aquatica;Prunella vulgaris*
	2	*Filipendula ulmaria;Potentilla anse*
	3	*Chamerion angustifolium;Anagallis*
	4	*Populus nigra;Cardamine pratensis*
	5	*Viburnum opulus;Adoxa moschatel*

;Stachys betonica
rina
arvensis;Lysimachia nummularia;Lysimachia vulgaris;Lysimachia nemorum
;Cardamine hirsuta;Populus tremula;Cardamine flexuosa;Nasturtium officinale;Sa
lina;Sambucus nigra

;Stachys betonica
rina
arvensis;Lysimachia nummularia;Lysimachia vulgaris;Lysimachia nemorum
;Cardamine hirsuta;Populus tremula;Cardamine flexuosa;Nasturtium officinale;Sa
lina;Sambucus nigra

lix repens;Salix viminalis;Salix caprea;Salix aurita;Alliaria petiolata;Mercurialis per

lix repens;Salix viminalis;Salix caprea;Salix aurita;Alliaria petiolata;Mercurialis per

ennis;Linum bienne;Salix cinerea;Salix purpurea

ennis;Linum bienne;Salix cinerea;Salix purpurea
